# Ultrafast MAS and Deuteration for High‐Resolution Proton Detected Solid‐State NMR of Intact Fungal Cell Walls

**DOI:** 10.1002/cbic.70574

**Published:** 2026-10-23

**Authors:** Alvis Zvirgzdins, Jana Petkus, Rihards Aleksis, Piotr Paluch, Justinas Ceponkus, Merve Kübra Aktan, Anitra Zile, Ats Kaldma, Mai‐Liis Org‐Tago, Ago Samoson, Alons Lends

**Affiliations:** ^1^ Latvian Institute of Organic Synthesis Riga Latvia; ^2^ Faculty of Medicine and Life Sciences University of Latvia Riga Latvia; ^3^ Center of Molecular and Macromolecular Studies Polish Academy of Sciences Lodz Poland; ^4^ Institute of Chemical Physics Faculty of Physics Vilnius University Vilnius Lithuania; ^5^ Department of Materials Engineering Biomaterials and Tissue Engineering Research Group KU Leuven Leuven Belgium; ^6^ Tallinn University of Technology Tallinn Estonia

**Keywords:** deuteration, fast magic‐angle spinning, fungal cell wall, proton detection, solid‐state NMR

## Abstract

Glycans are the main components of the fungal cell wall and play a central role in fungal pathogenesis, making atomic‐level structural and dynamic insight into these biopolymers essential for the development of new antifungal therapeutics. ^1^H‐detected solid‐state NMR (ssNMR) at fast magic‐angle spinning (MAS) offers an unprecedented opportunity to study glycan site, specifically at atomic resolution in intact fungal cell walls. However, the ^1^H resolution and sensitivity in ssNMR are often limited for detailed studies, especially for rigid and highly heterogeneous samples, such as intact fungal cells. Here, we conducted a detailed study on ^1^H line width and ^1^H *T*′_2_ coherence times in the rigid core of the human pathogenic *Candida albicans* fungal glycans at 60–150 kHz MAS rates and partial fungal cell wall deuteration schemes. We showcased the possibilities and limitations of ^1^H‐detected multidimensional ^1^H/^13^C chemical shift assignment experiments for rigid cell wall glycans. Furthermore, we probed dynamics using relaxation measurements, providing access to motions spanning from nanoseconds to microseconds. Overall, we demonstrated significant methodological advances in ^1^H‐detected ssNMR spectroscopy, achieving unprecedented ^1^H resolution of rigid glycans in intact fungal cell walls. These developments could be applicable for ssNMR detailed studies of intact cell walls in other human pathogens.

## Graphical abstract

The high structural complexity of fungal and yeast cells limits the applications of proton‐detected solid‐state NMR spectroscopy. Here, we conducted a detailed study of the dependence of MAS rates and deuteration levels on the proton line width and relaxation times in glycans from intact human‐pathogenic yeast cells. The achieved unprecedented ^1^H resolution at 150 kHz MAS for such heterogeneous systems enabled chemical shift assignment and site‐specific dynamics studies of glycans within an intact cellular environment.

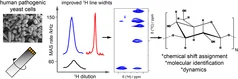

## 1 Introduction

Among the estimated 3 million fungal species, nearly 300 have been identified as human pathogens [1]. Invasive fungal infections caused by the genera *Aspergillus*, *Candida*, *Cryptococcus*, and *Pneymocystis* [2] are particularly dangerous to humans with compromised immune systems. In recent years, the number of infections and associated fatalities has risen due to the evolution of antifungal resistance in human pathogenic fungal species [3]. The fungal cell wall plays a major role in protecting fungi from environmental stresses and regulating the fungal life cycle, making it an attractive target for antifungals [4, 6]. Therefore, structural studies at the atomic level of intact fungal cell walls are crucial for the further development of new therapeutics, diagnostics, and gaining deeper knowledge about fungal pathogenesis. Previously, most studies of cell wall glycan structure relied on mass spectrometry/gas chromatography and liquid‐state NMR methods [7]. These techniques required the solubilization of cell walls under harsh chemical or biological conditions, which can disrupt their chemical composition and cellular integrity, leading to inconsistent outcomes. Recently, solid‐state NMR (ssNMR) spectroscopy at magic‐angle spinning (MAS) has emerged as a suitable nondestructive technique for analyzing molecular architecture, hydration, dynamics, and drug interactions in intact fungal cell walls [8, 9, 10]. The majority of these ssNMR studies on fungal cell walls were performed with ^13^C detection. Notably, despite the high structural complexity of intact fungal cells, the ^13^C line widths for glycans [11, 12] in ssNMR spectra were on the same order as reported for proteins [13, 14] with a high structural order. This allowed researchers to study the main cellular components based on ^13^C detection. To achieve a sufficient signal‐to‐noise ratio (SNR) in ^13^C‐detected multidimensional spectra, high amounts (10–40 mg) of the labeled sample are required, often limiting the wider applications of ssNMR for cellular research, particularly for fungi that require specific isotopes, conditions, and reagents to sustain growth in vitro conditions [15, 16]. Recent advances in ssNMR probe technologies have substantially increased MAS with smaller‐diameter ssNMR rotors [17], thereby significantly reducing the required sample amounts [18]. The decrease in SNR due to lower sample amounts can be compensated by direct detection of a high gyromagnetic ratio nucleus, ^1^H [19, 20]. Previously, ^1^H‐detected ssNMR spectroscopy across different MAS regimes has been applied to structural, dynamic, and interaction studies of a wide variety of biomolecules [21, 22]. Consequently, the achievements of ^1^H detection for single biomolecules have sparked interest in applying ^1^H detection to study cell walls in intact microorganisms and plants [23, 24, 25, 26, 27, 28, 29].

Intrinsic mobility contrast among fungal cellular components facilitates the selective rendering of molecular environments by ssNMR based on motional time scales [30, 31]. Flexible glycans, characterized using ^1^H–^13^C J‐coupling‐based transfers, exhibited ^1^H line widths of 30–200 Hz [23, 25, 32, 33], allowing high‐resolution structural studies. In contrast, rigid glycans probed via dipolar coupling, such as ^1^H–^13^C cross‐polarization (CP), yield significantly broader ^1^H line widths (500–1000 Hz at 40–105 kHz MAS) [25, 27, 29, 34] compared to aliphatic ^1^H resonances in proteins. This was due to a much higher degree of structural inhomogeneity in intact cellular samples. Despite limited ^1^H resolution, a few 2D and 3D ^1^H‐detected ssNMR assignments and dynamics studies have been demonstrated for rigid‐core glycans in intact bacteria, fungi, and plants [24, 27, 28, 29]. However, detailed studies of ^1^H line widths in ssNMR spectroscopy for rigid glycans in intact cellular samples are still lacking.

In ssNMR spectroscopy, the total ^1^H line widths (considering Lorentzian line shapes) consist of homogeneous and inhomogeneous contributions in the absence of chemical exchange [35, 36, 37, 38]. Using the Hahn‐echo [39] sequence, it is possible to determine ^1^H *T*′_2_ from which the homogeneous contributions to the line widths can be calculated as 1/*π* *T*′_2_. The homogeneous contributions can be reduced by increasing the MAS frequency, which leads to more effective averaging of ^1^H–^1^H dipolar couplings, the main contributors to the homogeneous part of ^1^H line widths [40]. The inhomogeneous part arises from the sample’s heterogeneity, which is especially prominent in intact cellular samples, and is also limited by probe shimming and magnetic field inhomogeneity [36, 41]. While heterogeneity arising from local disorder in single biomolecules can be mitigated by modifying sample preparation conditions [42], this approach is not fully applicable to whole organisms, where maintaining cellular integrity is essential. Another approach to reduce the homogeneous part of ^1^H line widths is to dilute the ^1^H–^1^H dipole coupling network by exchanging protons with deuterons. In proteins, the levels of ^1^H/^2^H exchange at labile and nonlabile sites can be tuned by varying the D_2_O:H_2_O ratio and/or by supplementing the sample preparation medium with deuterated carbon sources, cell extracts [43, 44], and deuterated amino acids [45]. Based on the available sample amounts and ssNMR spinning capabilities, the deuteration levels and sites can be adjusted [46, 47, 48] to leverage ^1^H resolution and SNR in multidimensional ssNMR spectra. In ^1^H‐detected ssNMR spectroscopy of glycans, only partial deuteration of the fungal cell wall is feasible, since C–H cross‐peaks are the main spectroscopic reporters. The ^2^H nuclei can be incorporated into the fungal cell wall matrix as C─D or O─D bonds by supplementing the cultivation medium with D_2_O and/or deuterated D‐glucose [49, 50]. The incubation of cellular samples in D_2_O can lead to H/D exchange of spatially water‐accessible, labile protons [51]. Deuterated samples enable the exploitation of the ^2^H quadrupolar parameters (e.g., the quadrupolar coupling constant *C*_Q_ and the asymmetry parameter *η*) to probe cell wall dynamics and water accessibility [52, 53]. For complex polysaccharides, ultra‐high magnetic fields (above 1 GHz) would also be beneficial for increasing peak resolution in heterogeneous biological systems with high polymorph content [25, 54, 55].

Previous work by the group of Prof. Wang demonstrated scalar‐ and dipolar coupling‐based ^1^H‐ and ^13^C‐detected ssNMR experiments to probe rigid and mobile glycans in *Candida albicans* (*C. albicans*) fungal cell walls [56, 57, 58, 59]. At 60 kHz MAS, the 2D CP hCH [58] spectrum revealed that the rigid core of the *C. albicans* cell wall is composed predominantly of β‐1,3‐glucan, and with around 5% chitin. Importantly, all six ^1^H–^13^C cross‐peaks of β‐1,3‐glucan were resolved in the 2D CP hCH spectrum, highlighting *C. albicans* as a suitable model system to study rigid core fungal glycans with ^1^H‐detected ssNMR spectroscopy. We selected the β‐1,3‐glucan polymer as the primary target for detailed ssNMR studies because it is the predominant structural component of the inner core in many fungal species [4]. Despite previous developments, the field of ^1^H‐detected studies of intact cells still lacks a systematic and quantitative assessment of ^1^H line widths, relaxation behavior as a function of MAS frequency, assignment robustness, dynamics, and the impact of partial deuteration. Addressing these gaps is essential for establishing broadly applicable ^1^H‐detected ssNMR methodologies to study glycans and other biomolecules in intact cells.

In this study, we explore in detail the dependence of ^1^H line widths and coherence lifetimes (*T*′_2_) on MAS frequency for protonated and deuterated *C. albicans* cell walls (strain SC5314) for β‐1,3‐glucan using CP‐based ^1^H‐detected ssNMR experiments. We demonstrate that both increased MAS frequencies and fungal cell wall deuteration techniques can substantially reduce ^1^H line widths and increase ^1^H *T*′_2_ relaxation times. Additionally, we showcased ^1^H and ^13^C chemical shift assignment possibilities and limitations using multidimensional proton‐detected spectra, and detailed dynamic studies using ^13^C *R*_1_ and *R*_1_ *ρ* relaxation rates for rigid glycans in fully protonated fungal cell walls.

## 2 Results and Discussion

### 2.1 Sample Preparation

Here, we adapted the previously published liquid YPD culture protocol [57] for small‐scale use with *C. albicans* yeast cells in 10 mL of liquid medium, which required only 100 mg of ^13^C D‐‍‍‍glucose and 100 mg of ^15^N ammonium chloride. The samples were incubated at 30 °C for 24 h with shaking at 200 RPM, followed by cell fixation with 2% paraformaldehyde for 24 h at 4 °C and 3 washes with H_2_O, yielding approximately 5 mg of labeled *C. albicans* cells from a 10‐mL liquid medium. The growth state of fungal/yeast samples can be adjusted to match the designed morphotype transition state for ssNMR studies in solid or liquid media [12, 34, 60]. Scanning electron microscopy (SEM) with backscattered electron (BSE) imaging at 10‐µm resolution showed yeast‐like cell images for *C. albicans* (Figure S1A,C), similar to images obtained with SEM of *C. albicans* cells grown for 3 days in protonated YPD medium [57]. The details of sample preparation are provided in the Supporting Information.

### 2.2 ^1^H Line Widths for Fully Protonated *C. albicans*

Figure 1A displays 2D CP hCH spectra acquired at 60 and 150 kHz MAS for the β‐1,3‐glucan region in UCN‐labeled *C. albicans*. In both spectra, ^1^H–^13^C correlations corresponding to C1–C6 sites are well resolved. The full 2D CP hCH spectrum at 150 kHz is shown in Figure S2A, and the NMR acquisition parameters for all NMR experiments are in Table S3. In addition to β‐‍‍‍1,3‐glucan peaks, we observed C–H correlation peaks originating from lipids and protein amino acids, as well as weak signals from chitin, in line with previously reported ssNMR data [58] for *C. albicans* samples (Figure S2A). At 60 kHz MAS, the average ^1^H line widths for H1–H6 protons in β‐‍‍1,3‐glucan were 652 ± 155 Hz (Figure 1C), consistent with values reported for 2D CP hCH experiments on fungal samples at MAS between 40 and 100 kHz. The similarity of ^1^H line widths across H1–H5 resonances (Figure S3) indicates comparable local structural heterogeneity among covalently bound protons within the β‐1,3‐glucan of the cell wall. The H6/H6′ protons of the –CH_2_–OH moiety in β‐1,3‐glucan exhibited narrower ^1^H line widths compared to H1–H5 protons, due to a higher degree of mobility, which averages out more of the ^1^H–^1^H dipolar contributions to ^1^H line widths. Site‐specific ^1^H *T*′_2_ measurements (fits shown in Figure S4A) yielded an average homogeneous line width of 453 ± 1 Hz at 60 kHz MAS. Increasing the MAS frequencies to 150 kHz resulted in a near‐linear increase in the bulk ^1^H *T*′_2_ coherence time (Figure 1B), consistent with trends observed for protons in CH sites, as previously reported for fully protonated proteins [22, 61], RNA [62], and small organic molecules [37, 63, 64, 65]. At 150 kHz MAS, the average total ^1^H line width of β‐1,3‐glucan decreased to 353 ± 60 Hz (Figure 1C), while the homogeneous contribution was reduced to 203 ± 60 Hz (calculated from ^1^H *T*′_2_, fits are shown in Figure S4B), corresponding to a ~55% narrower ^1^H line width. This order of total line width reduction is comparable to the 30%–70% decreases reported for protons from CH groups in fully protonated cellulose samples [66] and microcrystalline GB1 protein [61] and the viral capsid Cp149 [38]. For *C. albicans* cells, the ^1^H inhomogeneous contributions of HC protons were 63 ± 9 Hz, still significantly higher than the reported value for GB1 (~20 Hz) but closer to Cp149 (~126 Hz).

**FIGURE 1:**
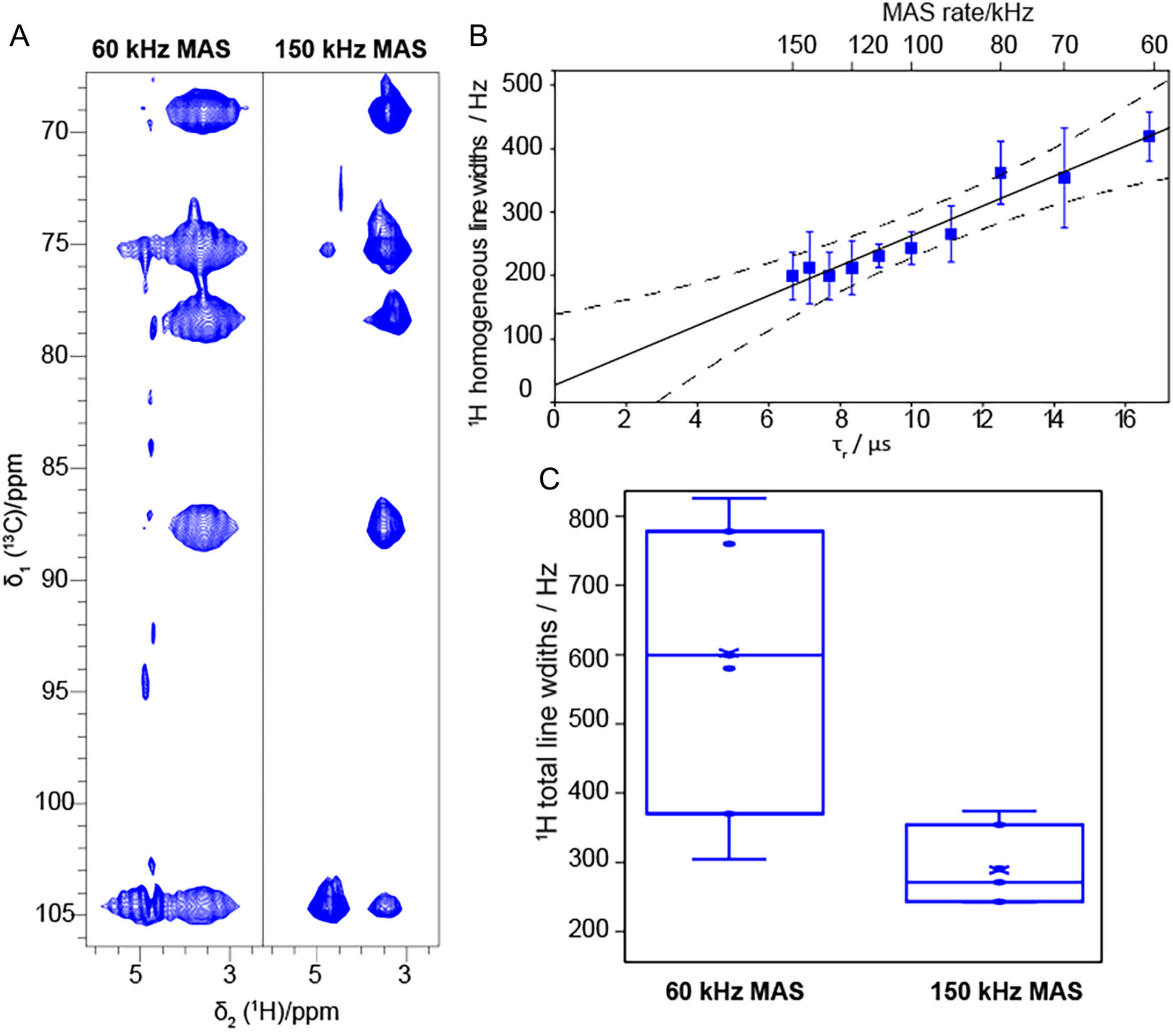
(A) 2D CP hCH spectrum at 60 and 150 kHz MAS for the glycan region of UCN *C. albicans*. (B) Dependence of the bulk homogeneous ^1^H line widths for β‐1,3‐glucan on MAS frequency/rotor period, fitted with a linear function (the 95% confidence interval is indicated by dashed lines). (C) ^1^H line width statistics at 60 and 150 kHz MAS. Boxplots were constructed from 6 individual ^1^H line width measurements; horizontal lines show medians, and *x* markers show means. Circles represent individual data points, and whiskers extend to the minimum and maximum values.

At 150 kHz MAS, the values of ^1^H line widths become more uniformly distributed compared to data acquired at 60 kHz MAS (Figures 1C and S3).

To probe highly dynamic components, we also acquired a 2D INEPT hCH spectrum at 150 kHz MAS (Figure S2B). Fewer CH correlation peaks were observed than in the same spectrum of intact *C. albicans* cells acquired at 13.5 kHz MAS in a 3.2‐mm rotor [58]. Thus, no gain with increasing MAS frequency was observed for the highly dynamic glycans of the *C. albicans* cell wall. Larger‐diameter rotors can accommodate more material and are better suited for probing highly flexible regions of the fungal cell wall, provided that a sufficient amount of the sample is available. Previously, higher SNR in 2D INEPT hCH spectra of flexible parts of Corynebacterial cell walls was demonstrated in a 1.3‐mm rotor acquired at 60 kHz MAS, compared to the same experiment acquired in a 0.7‐mm rotor at 100 kHz MAS [31].

### 2.3 Chemical Shift Assignment

Next, we investigated in detail a multidimensional ssNMR strategy for the ^1^H and ^13^C chemical shift assignment of β‐‍‍1,3‐glucan in the UCN *C. albicans* sample using 2D CP hCH (Figure 2A) and 3D CP hCCH (Figure 2B) and HcCH TOCSY (Figure 2C). Both 3D spectra were acquired with a 16‐ms WALTZ‐16 [67] isotropic ^13^C–^13^C mixing scheme (acquisition parameters in Table S4), which was previously used for protein side‐chain assignments [38, 68].

**FIGURE 2:**
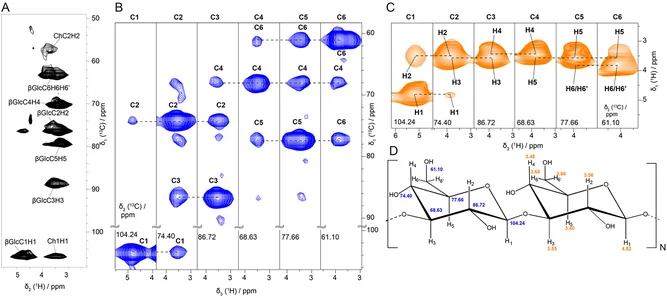
(A) The 2D hCH spectrum with ^1^H and ^13^C chemical shift assignments. (B) 2D ^1^H–^13^C correlation strips for C1–C6 sites in β‐1,3‐glucan. (C) The 2D ^1^H–^1^H strips for C1–C6 sites. With a dotted line, intramolecular linkages are indicated. (D) The molecular structure with ^1^H and ^13^C shifts assignments for β‐1,3‐glucan.

For each carbon resonance, we extracted a ^1^H–^13^C strip in which 2–3 CC correlations with preceding or subsequent carbons were resolved (Figure 2B). We started the chemical shift assignment by identifying the anomeric C1 shift at 103.4 ppm, which corresponds to the β‐anomer. For α‐anomers, C1 typically resonates 2–‍‍‍5 ppm upfield [69]. Glycosylic bond modifications in β‐1,3‐glucan were identified using ^13^C chemical shifts, which are strongly affected by substituent electronegativity and orientation [70]. We established sequential connections between adjacent carbons by tracing them through ^13^C‐resolved 2D ^1^H–^13^C strips, identifying all ^1^H and ^13^C chemical shifts in β‐1,3‐glucan (shown on the molecular structure of β‐1,3‐glucan in Figure 2D and Table S1). To compare the ^1^H‐detected assignment experiment with a conventional ^13^C‐detected approach, we acquired a 2D DARR spectrum [71] with 20‐ms mixing time at 12 kHz MAS and superimposed it with the 2D ^13^C–^13^C planes from the 3D hCCH TOCSY spectrum recorded at 150 kHz MAS (Figure S5). Both spectra exhibited similar spectral fingerprints and spin topologies of β‐1,3‐glucan. The 2D DARR spectrum with 20‐ms mixing time contained more ^13^C–^13^C cross‐peaks per resonance (Figure S5), as 16 ms J‐coupling mediated transfer experiments typically yield 2–‍3 bond correlations [72]. The spatial ^13^C–^13^C transfers CORD [73], PDSD [12], DREAM [74], and PARIS [75] recoupling sequences were applied for cellular samples, achieving similar 2–3 bond CC transfers efficiencies at slow (12–18 kHz) MAS. Although for UCN *C. albicans*, the 2D hCH and 3D hCCH spectra were sufficient to assign all ^13^C and ^1^H shifts of β‐1,3‐glucan, we additionally probed the ^1^H–^1^H correlations using a 3D HcCH TOCSY experiment [76]. The ^1^H–^1^H sequential correlations at specific carbons can be useful for ^1^H and ^13^C chemical shift assignment in structurally more complex glycans with various branching schemes and are widely used in liquid‐state NMR [77]. Figure 2C shows 2D ^1^H–^1^H strips for all ^13^C resonances of β‐1,3‐glucan. The anomeric H1 proton exhibited a distinct chemical shift (4.82 ppm) compared to other carbon‐bound protons in β‐1,3‐glucan, and the correlation between H1 and H2 sites was unambiguously identified (Figure 2A). Deciphering other proton resonances from ^1^H–^1^H correlations in the 3D HcCH spectrum was only possible with prior knowledge of ^13^C chemical shifts. The ^1^H chemical shift dispersion of H2–H6 sites in substituted glycans is inherently limited to a range of around 1.3 ppm [78], which also poses challenges for unambiguous ^1^H resonances even in liquid‐state NMR [77]. More unambiguous ^1^H–^1^H correlations for this UCN *C. albicans* sample could be identified by increasing ^1^H chemical shift dispersion at higher magnetic field strengths [79,‍80] and/or decreasing ^1^H line widths at higher MAS frequencies.

### 2.4 Polysaccharide Dynamics

The high ^1^H resolution and sensitivity achieved in the 2D CP hCH spectrum at 150 kHz MAS enabled site‐specific measurements of ^13^C *R*_1_ and *R*_1_ *ρ* relaxation rates for β‐1,3‐glucan in *C. albicans*, providing direct insight into the dynamic disorder in the cell wall. We estimated molecular correlation times for ^13^C *R*_1_ and *R*_1_ *ρ* ssNMR relaxation experiments using the “detectors” approach [81]. The corresponding simulated detector responses are shown in Figure S6A. At an 18.8 T magnetic field with 150 kHz MAS, ^13^C *R*_1_ rates primarily probe motions on the nanosecond timescale, whereas ^13^C *R*_1_ *ρ* rates acquired with a 13 kHz spin‐lock field are sensitive to microsecond‐timescale dynamics [82]. For the endocyclic C1–C5 sites, the measured ^13^C *R*_1_ values fall within a narrow range of 0.18–0.28 s^−1^ (Figure 3A, fits are shown in Figure S7A), while the corresponding *R*_1_ *ρ* rates span 18.4–21.3 s^−1^ (Figure 3B, fits are shown in Figure S7B), indicating comparable motional amplitudes on both ns and μs timescales across the pyranose ring carbons. The observed variability in ^13^C *R*_1_ *ρ* rates is consistent with values reported for β‐ and α‐1,3‐glucans and chitin in the cell walls of *Cryptococcus neoformans* [27], *Aspergillus fumigatus*, *Rhizopus* *delemar* [29], and *Trichoderma harzianum* [83] measured at 60–100 kHz MAS. In contrast to the C1–C5 sites, the C6 site exhibits markedly enhanced mobility, with ^13^C *R*_1_ and *R*_1_ *ρ* rates approximately fourfold and twofold higher, respectively, than those of the C1–C5 sites (Figure 3A,B), reflecting strongly increased rotational freedom of the exocyclic C6 site.

**FIGURE 3:**
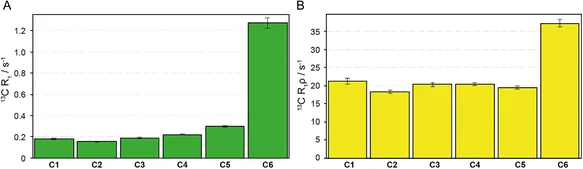
The β‐1,3‐glucan carbon‐specific ^13^C relaxation rates ((A) *R*_1_, (B) *R*_1_ *ρ*) for UCN *C. albicans* acquired at 150 kHz MAS. The error bars for relaxation rates were from relaxation‐time errors, expressed as 95% confidence intervals derived from the uncertainty of the exponential fit to individual decay curves.

The enhanced ^13^C *R*_1_ and *R*_1_ *ρ* relaxation rates for the C6 site can be attributed to rapid reorientations of the –CH_2_OH moieties, which have a higher degree of freedom, as previously reported in ssNMR experiments of other solid polysaccharides [52, 84]. The detector analysis revealed that motions at sites C1–C6 are detected on both near‐nanosecond (detector *ρ*_1_) and near‐microsecond (detector *ρ*_2_) timescales, whereas slower motions (detector *ρ*_0_) are observed only at sites C2–C5 (Figure S6B). This indicated collective motions at the C2–C5 sites on the microsecond timescale. In contrast, trans‐gauche reorientation is the dominant motion at site C6, and this site does not participate in collective motions. The C1 site, which is involved in glycosidic bond formation and is an anomeric carbon, exhibits faster dynamics than the collective motions of the core sites.

### 2.5 Deuterium Incorporation

Next, we prepared two deuterated *C. albicans* samples to study the ^1^H line widths of β‐1,3‐glucan in the cell wall. The first sample was produced using the inverse fractional deuteration approach (iFD) [35, 85, 86], in which ^2^H, ^13^C‐labeled D‐glucose served as the sole source of incorporation of ^2^H nuclei (iFD *C. albicans*). The second deuterated sample was produced using ^2^H,^13^C D‐glucose in a YPD growth medium containing 50% of D_2_O (50% DCN *C. albi‍‍cans*). Both deuterated samples were obtained in a deuterium‐containing cultivating medium without prior cell adaptation. We observed no major differences in growth curves between deuterated and protonated samples, as both reached the same lag phase (Figure S8). Fungal cell growth kinetics can vary among fungal species and depend on the growth medium and conditions used [87]. For some fungal species, adaptation to deuterated media may require several steps, with a gradual increase in deuterium content in solid or liquid growth media [29]. BSE imaging under magnetic immersion mode showed similar surface morphology for UCN and 50% DCN *C. albicans* cells (Figure S1A–D), indicating no major changes in fungal morphology upon incorporation of ^2^H nuclei in fungal cellular matrix.

To confirm the incorporation of deuterium as C─D and C─O─D bonds in the iFD *C. albicans* sample, we acquired a ^2^H spectrum (Figure 4A) at 10 kHz MAS and compared it with the same spectrum of pure ^2^H,^13^C‐labeled D‐glucose (Figure 4B). Two different ^2^H environments were distinguished in the ^2^H spectrum of iFD. One of the ^2^H sites exhibited a minor residual quadrupolar interaction, indicating a very mobile ^2^H environment. Thus, we assigned this to HDO, which was one of the products of ^2^H‐labeled D‐glucose yeast metabolism. The second ^2^H environment has a significantly larger quadrupolar interaction with *C*_Q_ = 89 ± 2 kHz and $\eta =0.98_{-0.06}^{+0.02}\;\pm \;2$, arising from ^2^H nucleus incorporation in C─D or C─O─D bonds in the fungal cell wall matrix. In uniformly labeled (^2^H, ^13^C) D‐glucose powder, the mean quadrupolar coupling ${\overset{―}{C}}_{Q}=161.5\;\pm \;0.5$ kHz and $\eta =0.01_{-0.01}^{+0.02}$. All ^2^H *C*_Q_ and *η*_Q_ values are shown in Table S2.

**FIGURE 4:**
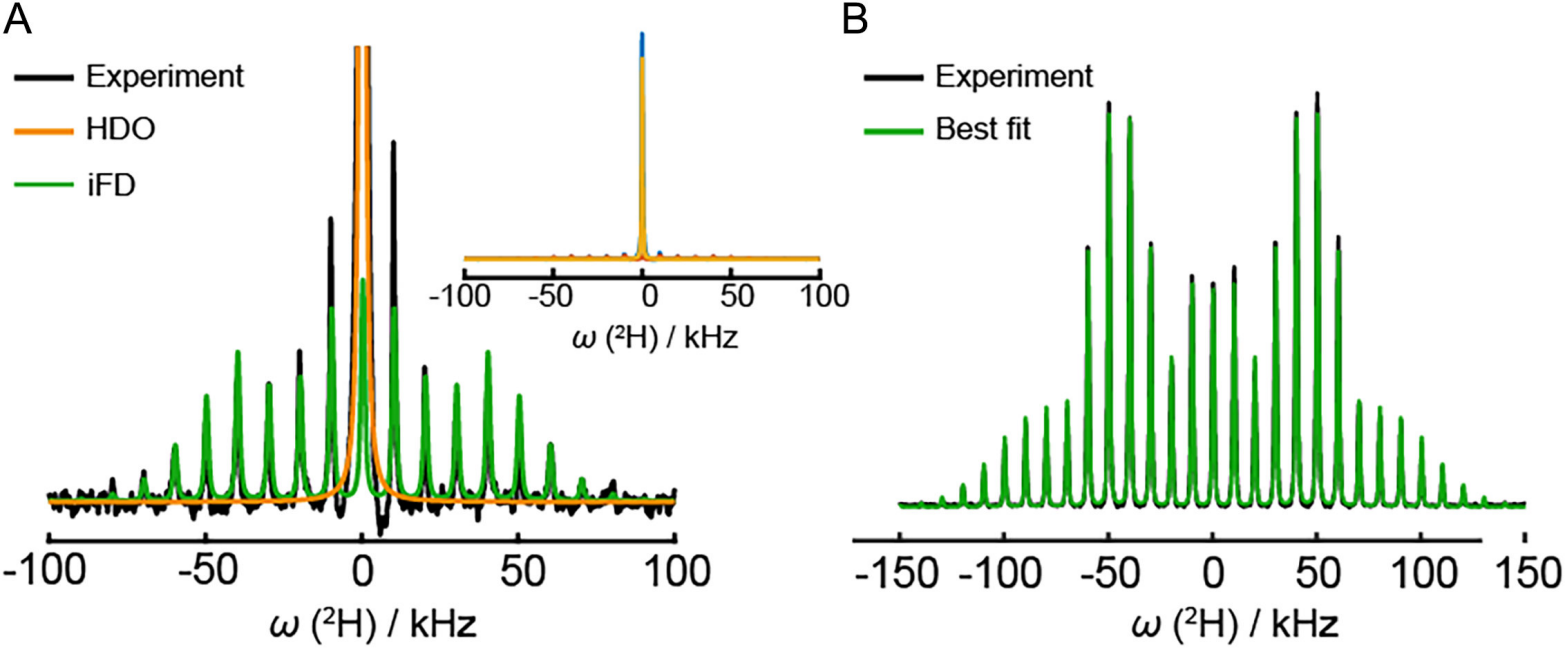
^2^H‐detected ssNMR spectra acquired at 10 kHz MAS: (A) iFD *C. albicans*. (B) ^2^H,^13^C‐labeled D‐glucose powder. In panel (A), the inset shows the entire ^2^H spectrum.

The smaller ^2^H average quadrupolar coupling constants ${\overset{―}{\text{C}}}_{Q}$ in the iFD *C. albicans* fungal sample can be attributed to an increased polysaccharide mobility [52] within the *C. albicans* cell wall compared with the structurally much more rigid ^2^H,^13^C D‐glucose microcrystalline powder.

Furthermore, the incorporation of covalently bound deuterium into carbon in *C. albicans* samples was confirmed by attenuated total reflection Fourier‐transformed infrared (ATR‐FTIR) spectroscopy [88]. Both deuterated *C. albicans* samples (iFD and 50% DCN) used in this study exhibited a characteristic C─D bond stretching peak around 2300 cm^−1^ [89, 90] in the ATR‐FTIR spectra, whereas this peak was absent in the nondeuterated samples (Figure S9).

### 2.6 ^1^H Line Widths for the Deuterated *C. albicans*

The 2D CP hCH spectra of iFD and 50% DCN *C. albicans* samples acquired at 60 kHz MAS (the overlay is shown in Figure 5A). The same ^1^H–^13^C crosspeaks of β‐1,3‐glucan as for the UCN sample were observed, indicating no substantial structural reorganization of the rigid component of *C. albicans* cell walls. At 60 kHz MAS, the total proton line widths for the iFD *C. albicans* sample were 335 ± 65 and 208 ± 34 Hz for the 50% DCN *C. albicans* sample (Figure 5B), substantially lower compared to fully protonated UCN *C. albicans* (652 ± 155 Hz). Previously, Yarava et al. [29] reported a 25%–50% reduction of total ^1^H line widths at 60 kHz MAS for α‐1,3‐glucan in the *A. fumigatus* cell wall, which was grown in D_2_O. Notably, the diastereotopic H6 and H6′ protons for the iFD *C. albicans* sample can be unambiguously resolved already at 60 kHz MAS compared to the same spectral region for the UCN sample (Figure S10A,B). At a 150 kHz MAS rate, the ^1^H line widths of 50% DCN DCN *C.* *albicans* sample decreased to 126 ± 34 Hz and the homogeneous contribution to 64 Hz (Figure S11, the ^1^H *T*′_2_ fits are shown in Figure S4C). The total ^1^H line width reduction was 44% compared to the fully protonated UCN sample at 150 kHz MAS. The inhomogeneous contributions of the ^1^H line widths remained approximately the same in both the 50% DCN (69 ± 14 Hz) and UCN (63 ± 9 Hz) samples (Figure S11). For the 50% DCN sample, inhomogeneous contributions are still dominating the total ^1^H line widths.

**FIGURE 5:**
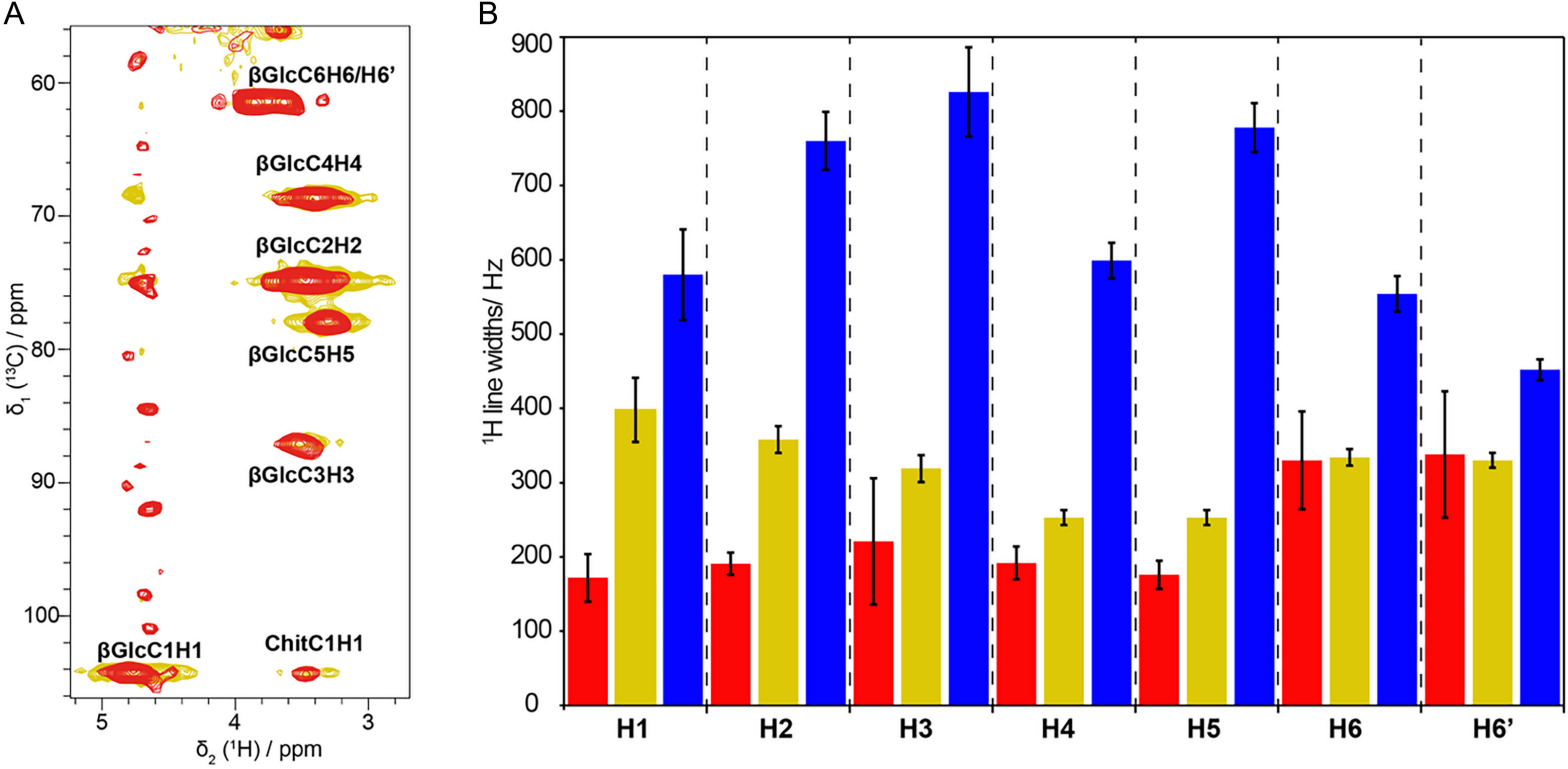
(A) 2D CP hCH spectral overlay at 60 kHz MAS for iFD (yellow) and 50% DCN (red) *C. albicans* samples. (B) Total site‐specific ^1^H line width statistics for UCN (blue), iFD (yellow), and 50% DCN (red) at 60 kHz MAS. The error bars for ^1^H line width were calculated from ^1^H *T*′_2_ relaxation time errors, expressed as 95% confidence intervals derived from the uncertainty of the exponential fit to individual decay curves.

We also explored partial deuteration by incubating a UCN sample in 500 μL of D_2_O for 3 days, allowing the exchange of spatially accessible labile protons. At 40 kHz MAS, the ^1^H line widths for the back‐exchanged sample in the 2D CP hCH spectrum were around 900 ± 200 Hz (^1^H *T*′_2_ 0.59 ± 0.07 ms), compared to around 1000 ± 200 Hz (^1^H *T*′_2_ 0.47 ± 0.06 ms), indicating a slight improvement in ^1^H line widths and ^1^H *T*′_2_ times after D_2_O incubation after cell fixation. In cells, water accessibility to inner cellular compartments depends on the organism and the sample preparation for NMR studies. An additional advantage of sample incubation in D_2_O is the reduction of the water signal, as the water peak diminished and C–OH–water correlations were absent in 2D CP hCH spectra (Figure S12).

The incorporation of ^2^H nuclei can lead to deuterium isotope effects, thereby altering ^13^C resonances and complicating database‐driven ^13^C chemical shift assignments. In previous studies, the deuterium isotope effect on ^13^C shifts at C–OD sites in glycans was reported to be 0.03–0.09 ppm [91], whereas at C–D sites it was 0.33–0.39 ppm [92]. The average ^13^C line widths of the iFD and 50% DCN samples in the 2D CP hCH spectra were approximately 0.88 ppm. Consequently, we were unable to detect any deuterium isotope effects on the ^13^C chemical shifts in the deuterated *C. albicans* samples. We noted that a reduction in ^1^H line widths for the iFD sample led to a decrease in ^1^H signal intensity in 1D hCH experiments by approximately a factor of 3.5 relative to a fully protonated sample (Figure S13), due to lower ^1^H spin content. However, because deuterated samples exhibit significantly narrower ^1^H line widths even at 60 kHz MAS compared to fully protonated samples, deuterated fungal samples can be packed in larger‐volume rotors, thereby increasing the SNR in multidimensional spectra while maintaining sufficiently narrow ^1^H line widths at slower (40–60 kHz) MAS rates [48]. The optimal ^1^H‐detection experimental conditions for ssNMR studies of rigid cell walls depend on equipment availability and the feasibility of sample preparation. For 1.3–1.9‐mm MAS probes, partial deuteration with D_2_O or deuterated glucose is recommended, as sensitivity remains sufficient and deuteration significantly improves ^1^H line widths. In contrast, for 0.51–0.7 mm probes operating at 100–160 kHz MAS, fully protonated samples are preferable to achieve sufficient SNR and better ^1^H resolution. Smaller (0.51–0.7 mm) ssNMR MAS rotors are also particularly advantageous for samples available only in submilligram quantities, such as a single colony from the growth plates of clinical isolates.

## 3 Conclusion

We demonstrated that increasing MAS frequencies to 150 kHz linearly reduces ^1^H line widths and increases ^1^H *T*′_2_ coherence lifetimes of β‐1,3‐glucan within the rigid core of the highly heterogeneous *C. albicans* cell wall samples, reaching ^1^H line widths similar to protein ssNMR.

The combination of 2D CP hCH and 3D hCCH TOCSY experiments provides sufficient sequential connectivity for complete ^1^H and ^13^C chemical shift assignment for β‐1,3‐glucan. Comparison with conventional ^13^C detection methods exhibited similar spectral fingerprints, highlighting ^1^H detection in ssNMR as a comparable alternative for glycan identification.

The measured site‐specific glycan ^13^C *R*_1_ and *R*_1_ *ρ* relaxation rates revealed comparable dynamics on the ns and μs time scales for endocyclic C1–C5 sites, whereas C6 showed enhanced mobility due to rapid reorientation of the –CH_2_–OH group.

Fungal cell deuteration is an effective technique for improving ^1^H line widths without perturbing the native structural organization of fungal cells. Both iFD and 50%DCN labeling schemes improved ^1^H resolution; however, the overall sensitivity in ^1^H‐detected ssNMR spectra was decreased. MAS frequencies exceeding 150 kHz will benefit fully protonated samples, whereas partial cell deuteration is needed for ^1^H‐detected ssNMR studies at lower MAS frequencies. In addition, postsample incubation with D_2_O provides only a modest improvement in ^1^H line widths, suggesting limited D_2_O accessibility within the inner cellular compartments. Nevertheless, D_2_O incubation can suppress the water signal, simplifying the spectral analysis. Overall, the ^1^H‐detected ssNMR workflow established here for chemical shift assignments and dynamics studies of intact rigid *C. albicans* cell walls should be readily transferable to other fungal species with more structurally complex rigid cell walls. The improved spectral resolution and sensitivity provided by ^1^H‐detected ssNMR spectroscopy for studying such complex intact cellular systems will be further enhanced by recently emerged ultra‐high‐field NMR spectrometers, which provide increased chemical‐shift dispersion in both ^1^H and ^13^C dimensions [93] and, in combination with faster (above 160 kHz) MAS, extended ^1^H *T*′_2_ times. These developments could enable high‐resolution ^1^H‐detected ssNMR spectroscopy for a broader range of whole‐cell samples.

## Funding

This study was supported by the Latvijas Zinātnes Padome (2023/1‐0312).

## Conflicts of Interest

The authors declare no conflicts of interest.

## Supporting information

Supplementary Material

## Data Availability

The experimental details for this work are available in the Supporting Information. The ssNMR datasets and simulations are deposited in the Zenodo repository (https://doi.org/10.5281/zenodo.22955076).
