# Supplementary material for: Ultrafast MAS and Deuteration for High‐Resolution Proton Detected Solid‐State NMR of Intact Fungal Cell Walls
Source: Chembiochem. 2026 Oct 23;27(20):e70574. doi: 10.1002/cbic.70574 (PMC13691519; doi:10.1002/cbic.70574)
Supplement: Supplementary file 1 [file CBIC-27-e70574-s001.pdf]

Supporting Information

Ultra-Fast MAS and Deuteration for High-Resolution Proton Detected Solid-State NMR of Intact Fungal Cell Walls

Alvis Zvirgzdins[1,2], Jana Petkus[1,2], Rihards Aleksis[1], Piotr Paluch[3], Justinas Ceponkus[4], Merve Kübra Aktan[5], Anitra Zile[1,2], Ats Kaldma[6], Mai-Liis Org-Tago[6], Ago Samoson[6], Alons Lends[1]

[1]Latvian Institute of Organic Synthesis, Aizkraukles 21, Riga, Latvia

[2]Faculty of Medicine and Life Sciences, University of Latvia, Jelgavas Street 1, LV-1004, Riga, Latvia

[3]Centre of Molecular and Macromolecular Studies, Polish Academy of Sciences, Sienkiewicza 90-363, Lodz, Poland

[4]Institute of Chemical Physics, Faculty of Physics, Vilnius University, Sauletekio 3, LT-10257, Vilnius, Lithuania

[5]KU Leuven, Department of Materials Engineering, Biomaterials and Tissue Engineering research group, Kasteelpark, Arenberg 44, box 2450, 3001, Leuven, Belgium

[6]Tallin University of Technology, Ehitajate tee 5, 19086 Tallinn, Estonia

Sample preparation

Preparation of YPD minimal medium

0.19 g of YPD powder was dissolved in 100 mL of H_2O and autoclaved. The minimal medium for labeled isotopes was prepared by dissolving 0.019 g of YPD minimal medium broth powder in 100 mL of water, autoclaving it, cooling it to room temperature, and then adding 0.1 g D-glucose and 0.1 g NH_4Cl. The YPD medium was filtered through a 0.2 micron filter. The medium containing 50% D_2O was not autoclaved.

Fungal sample preparation

The slant of *C. albicans* (strain SC5314) was streaked onto a YPD agar plate, which was incubated for 16 h at 30 ^oC. A single colony was picked and resuspended in 10 mL of YPD minimal medium. The samples were incubated for ~20 h at 30 ^oC with shaking at 200 RPM in a Biosan environmental shaker-incubator ES-20.

Growth curves

A single *C. albicans* colony was resuspended in 4 mL of YPD minimal medium containing 1% NH_4Cl and 1% D-glucose, and the culture was grown overnight at 30 ^oC with shaking

at 200 RPM. After incubation, the resulting suspensions were divided into five aliquots and centrifuged at 14000 RPM for 3 min. The formed pellets were washed twice with YPD minimal medium. The supernatant was diluted in the appropriate medium until an OD_{600} value of 0.100, and the resulting suspension (150 μL) was pipetted into a 96-well Greiner flat-bottom microplate. We determined fungal growth by measuring absorbance at 600 nm at 10 min intervals over an 18 h period using *BMG Labtech ClarioStar plus* microplate reader set to 30 °C with orbital shaking at 200 RPM. The results were being shown with OD_{600} values corrected by subtracting the mean blank measurement (either YPD minimal medium or YPD broth).

Sample packing

The samples were packed using a home-made filling tool for the 0.51 mm rotors and the spiNpack tool (from Giotto biotech)[1] for the 1.3 and 3.2 mm rotors. The homemade filling tool for 0.51 mm rotors was spun 2 times for 40 min at 10k g and 4 °C in an Optima XL centrifuge. The spiNpack tool was spun for 1 h at 70k g and 5 °C in a Hitachi CP 80NX ultracentrifuge.

NMR spectroscopy

All ssNMR spectra were acquired on an 18.8 T Bruker NMR magnet. A 2D DARR spectrum with 20 ms mixing time was acquired using a 3.2 mm HCN Bruker E-free probe at 12 kHz MAS, with a probe temperature of 268 K, the sample temperature was around 279 K. The ^{1}H-detected spectra at 60-150 kHz MAS were acquired using a 0.51 mm HCN probe made by Dr. Ago Samoson (Darkland OU, Estonia) or a Bruker HFX 1.3 mm probe (at 40 and 55.555 kHz MAS). The bulk and site-specific ^{1}H T'_2 relaxation times have been extracted from 1D and 2D CP hCH experiments, acquired with ^{1}H Hahn-echo before the first ^{1}H-^{13}C CP transfer. The detailed ^{1}H-detected ssNMR experimental parameters for 3D assignment experiments are summarized in Tables S3 and S4. The sample's temperature at 150 kHz MAS was around 298 K, determined from the H_2O chemical shift[2].

The single pulse ^{2}H spectra were acquired using a 3.2 mm H/F X Bruker probe at 10 kHz MAS, with the probe temperature set to 268 K, the sample temperature was around 279 K.

All ^{1}H detected spectra were analysed using CcpNmr 2.5.2[3] and TopSpin 3.5. For the ^{1}H line widths measurements from 2D CP hCH spectra, no signal apodization functions were

applied in the ^{1}H dimension. The ^{1}H line widths were determined using CcpNmr 2.5.2. In cases of strong signal overlap, ^{1}H line widths were extracted from 1D projections of the 2D hCH spectra at the half maximum of the peak. In order to extract the quadrupolar tensor components, we fitted the ^{2}H spectra using SIMPSON simulated spectra following a procedure described previously[4]. The ^{2}H NMR spectra in SIMPSON[5] were simulated using Zaremba[6], Conroy[7] and Wolfsberg[8] scheme with 376 crystallites and 32 γ-angles. The HDO signal was modeled as a Lorentzian line shape.

The NMR relaxation data were analyzed using Bruker Dynamics Center software.

The detector responses for each carbon nucleus in β-1,3-glucan were simulated using pyDIFRATE scripts (https://github.com/alsinmr/pyDR). As input data, site-specific ^{13}C R_1 and $R_{1\rho}$ relaxation rates, MAS 150 kHz, amplitude of motion (S^2) 0.85 for C1-C5 and 0.5 for C6, ^{13}C spin lock field of 13 kHz in an 18.8 T NMR magnet.

ATR-FTIR spectroscopy

Samples in H_2O were placed on a diamond ATR crystal in a Bruker Alpha FTIR spectrometer. Samples were air-dried until no traces of H_2O absorption bands were observed in the spectra. All spectra were acquired at room temperature in the 4000-400 cm^{-1} spectral range using 2 cm^{-1} spectral resolution with 64 scans.

Scanning electron microscopy

The cells were pelleted and washed once with 1x PBS buffer (137 mM NaCl, 2.7 mM KCl, 10 mM Na_2HPO_4, 1.8 mM KH_2PO_4, pH=7.4), then gently resuspended in 0.1 M cacodylate buffer (pH=7.4) with 2.5% glutaraldehyde and incubated for 30 min. Afterward, the cells were washed once in 0.1 M cacodylate buffer and three times in 1x PBS by centrifugation at 1500 rpm for 5 min at +4°C. The cells were then dehydrated with an ethanol gradient (30%, 50%, 70%, 90%, 95%) for 20 min at each step, followed by three immersions in absolute alcohol for 20 min. The samples were left to air dry overnight.

The fixed cells were analyzed using SEM (NovaNanoSEM 450, FEI) under high-vacuum conditions with back-scattered electron (BSE) imaging, acquired in magnetic immersion mode. A low accelerating voltage of 5 keV was used to reduce beam-induced damage to the *C. albicans* cells. The working distance and spot size were set to 5 and 3 mm, respectively. Prior to imaging, the samples were coated with a 5 nm layer Pt/Pd coating (Q150TS, Quorum) to minimize surface charging.

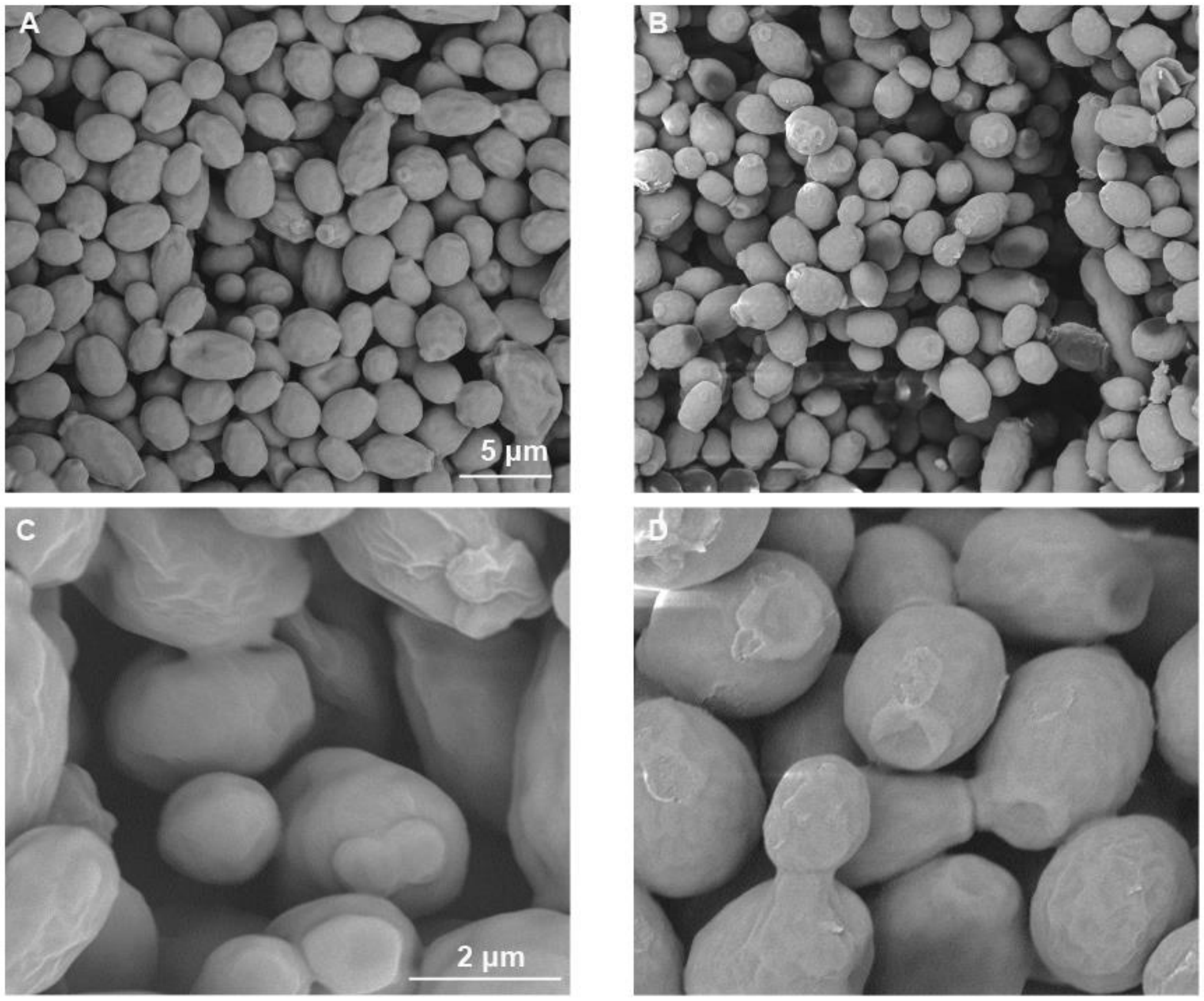

Figure S1. BSE images of *C. albicans* cells on UCN labeled sample surfaces(A,C) and (B,D) 50%DCN labeled surfaces, acquired under magnetic immersion mode at x10,000 magnification (A,B) and x40,000 magnification (C,D).

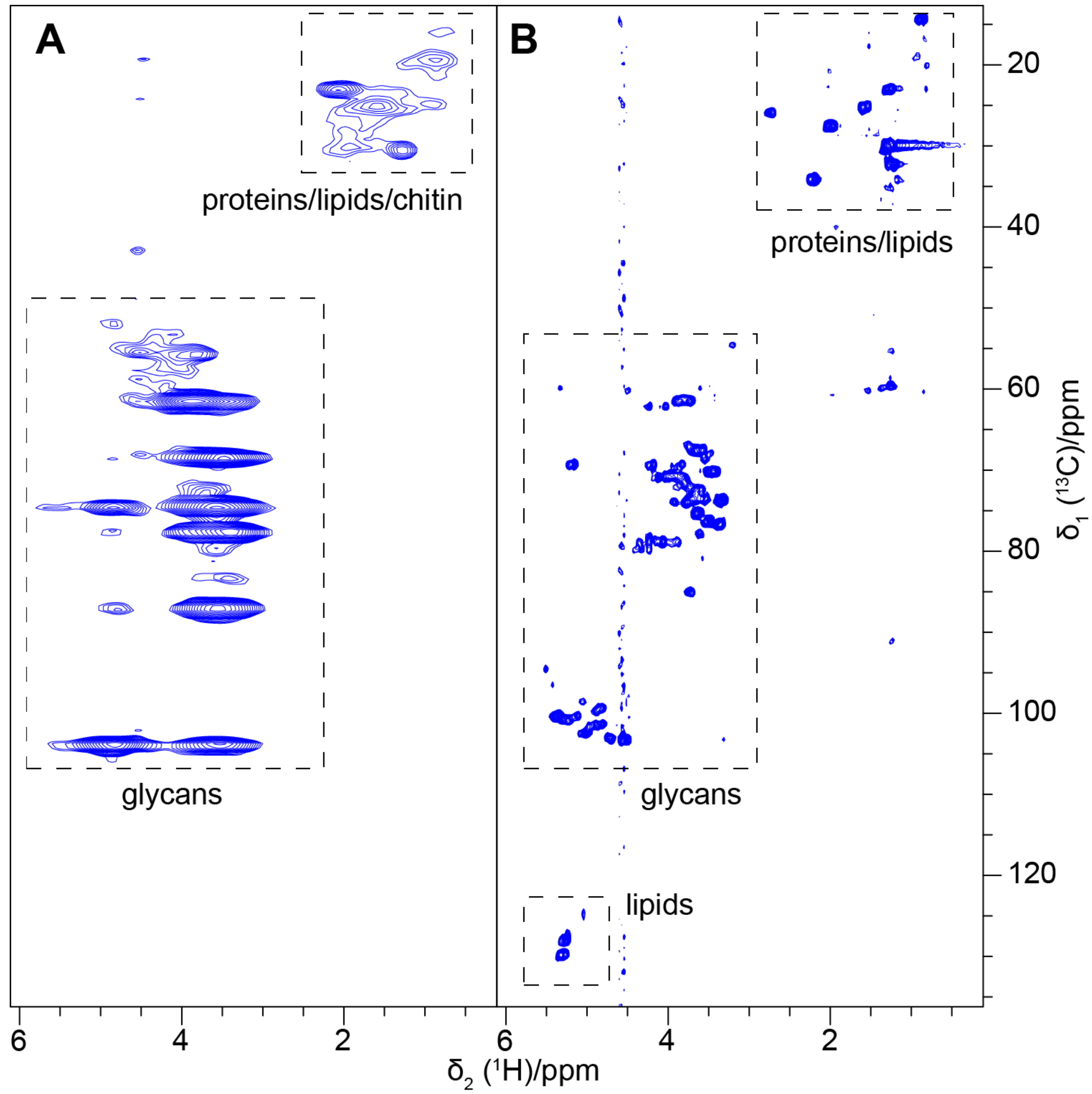

Figure S2. The full 2D CP (A) and INEPT (B) hCH spectra of UCN *C. albicans* sample acquired at 150 kHz MAS.

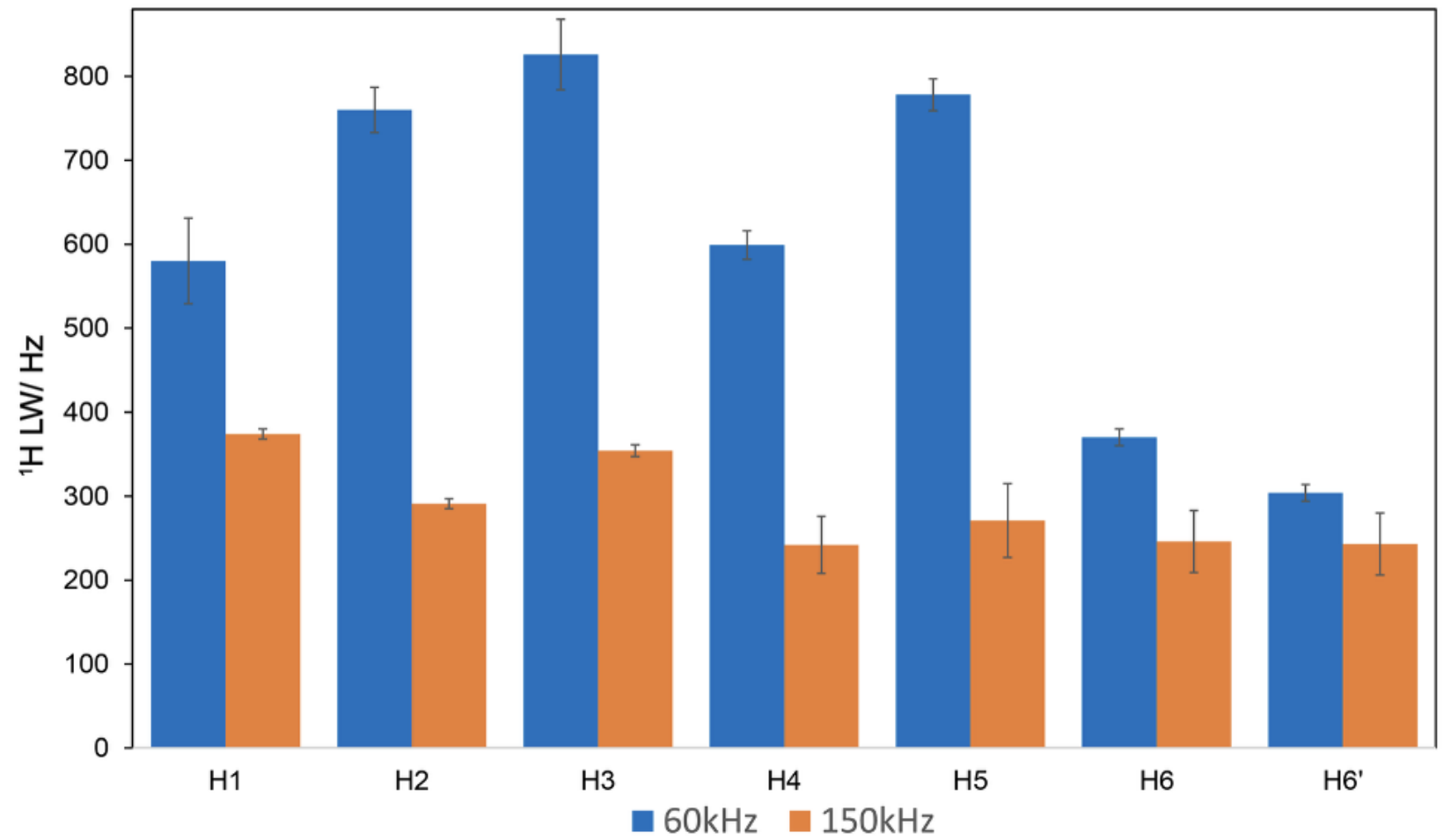

Figure S3. The site-specific total ^{1}H line widths for β-1,3-glucan in UCN *C. albicans* at 60 (blue) and 150 kHz (orange) MAS. All relaxation time errors were expressed as 95% confidence intervals derived from the uncertainty of the exponential fit to the individual decay curves. Relative ^{1}H T'_2 relaxation time errors were used to calculate errors in ^{1}H line width.

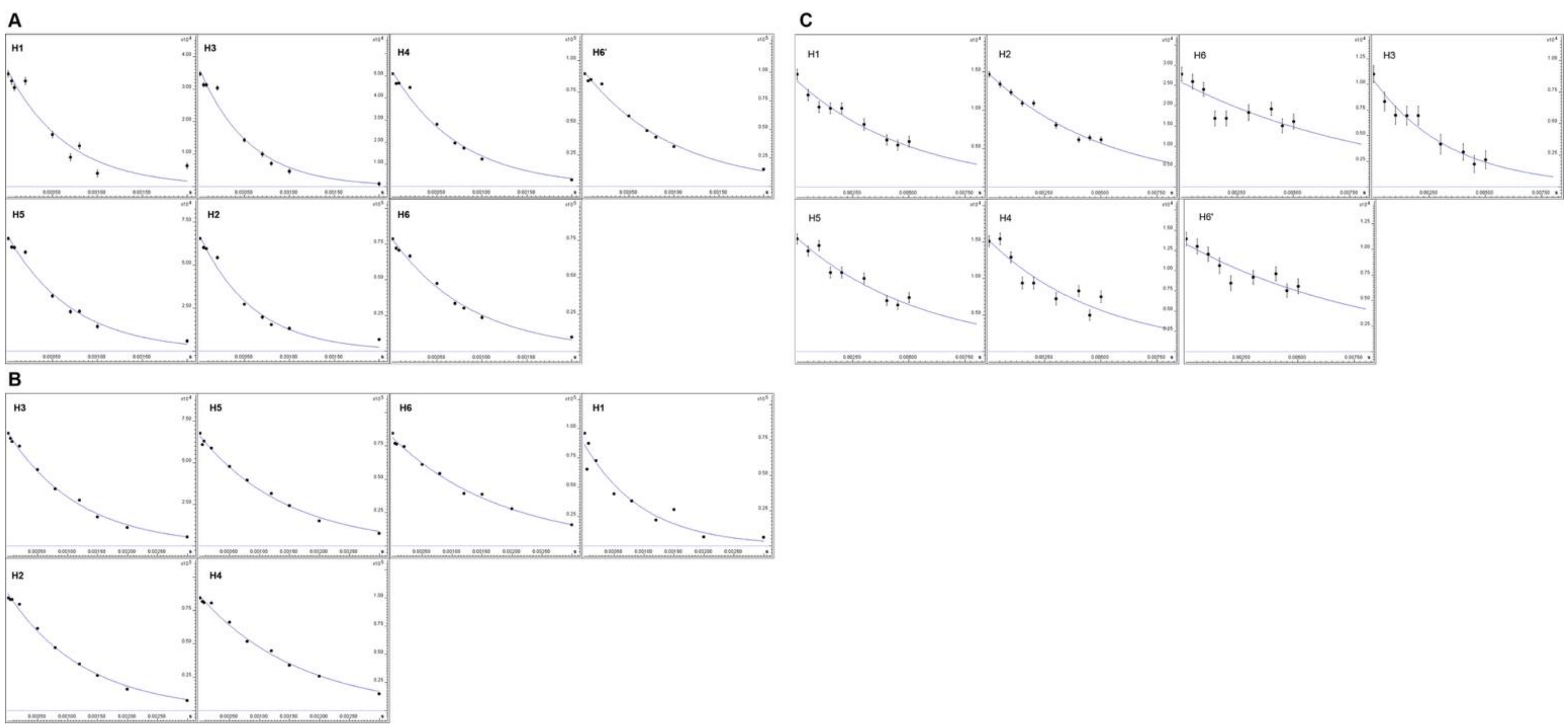

Figure S4. The site-specific ^{1}H T'_2 relaxation time decays with mono-exponential fits for β-1,3-glucan in *C. albicans*. A - UCN 60 kHz MAS, B - UCN 150 kHz, C - 50%DCN 150 kHz MAS.

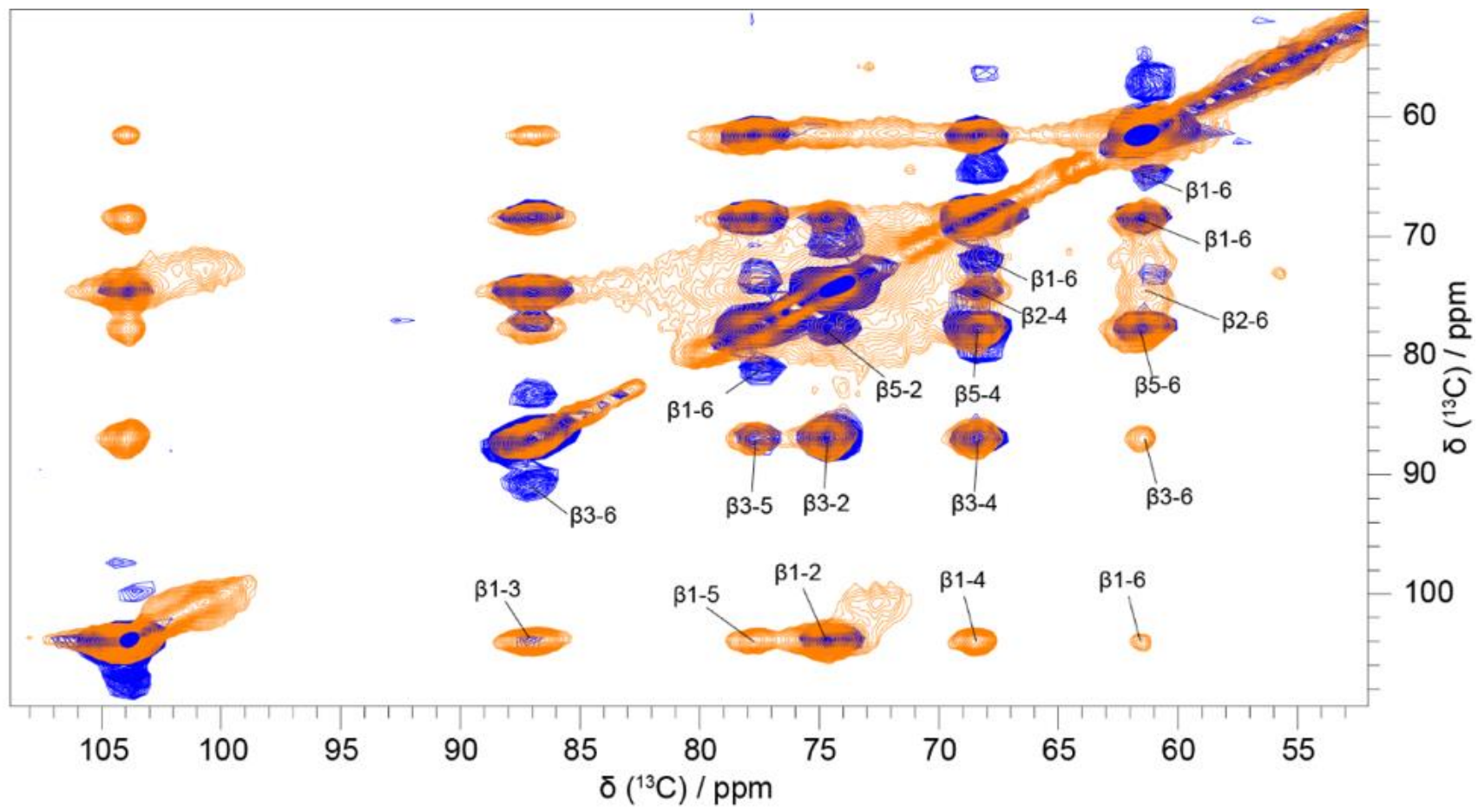

Figure S5. The overlay of a 2D DARR spectrum with 20 ms mixing (orange) with a 2D CC projection (blue) from a 3D hCCH spectrum with a 16 ms TOCSY transfer.

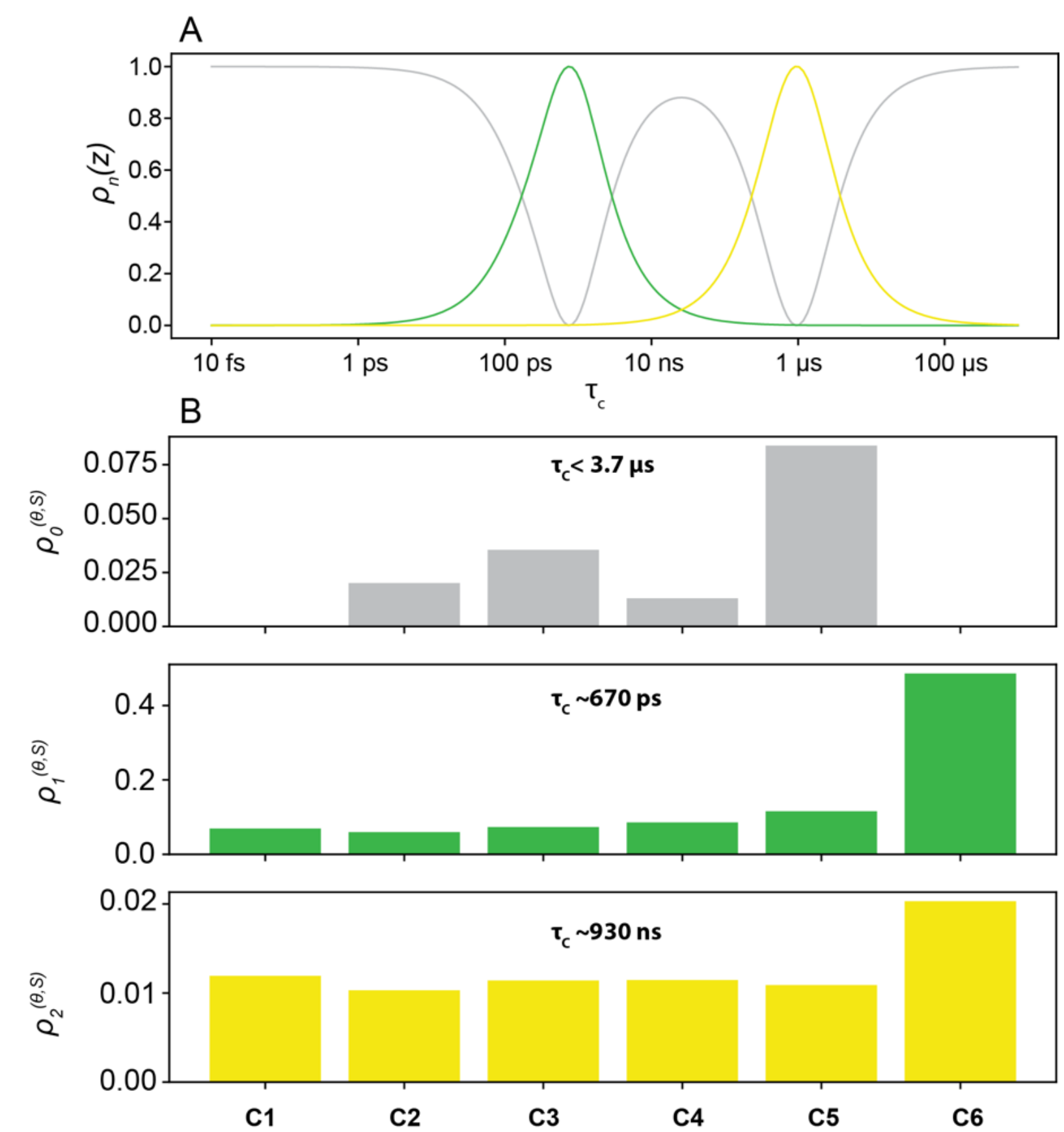

Figure S6. A - The “detectors” responses for ^{13}C R_1 (green) and $R_{1}\rho$ experiments (13 kHz ^{13}C spin lock) at 150 kHz MAS, 800 MHz NMR magnet. B - Site-specific ρ_0, ρ_1, and ρ_2 “detector” responses for different τ_c times (ρ_0 detector responses are calculated using only initial parameters of the ^{13}C nucleus, and it is not based on experimental relaxation data).

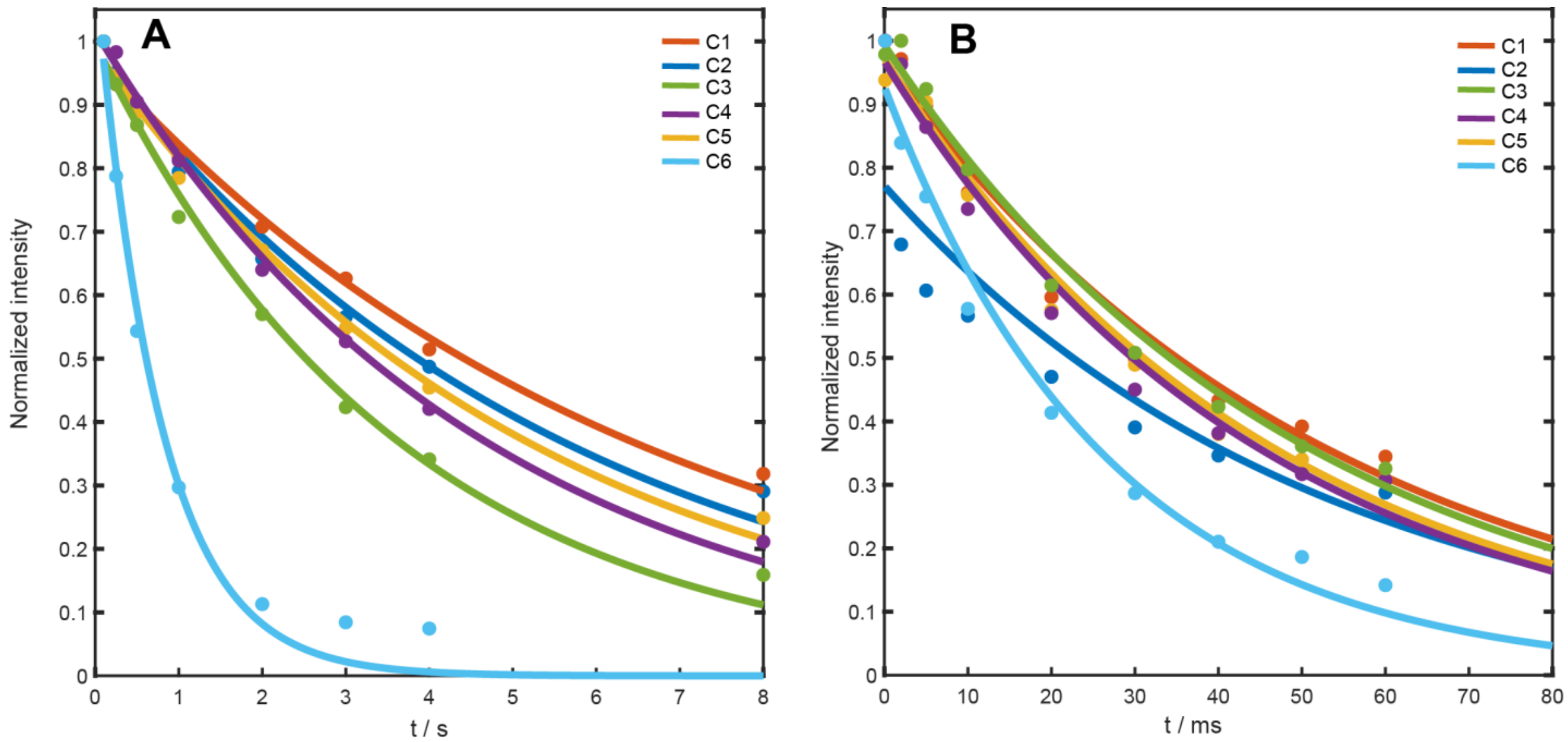

Figure S7. Site-specific mono-exponential ^{13}C relaxation time fits for β-1,3-glucan, A - T_1 relaxation, B - $T_{1\rho}$ relaxation.

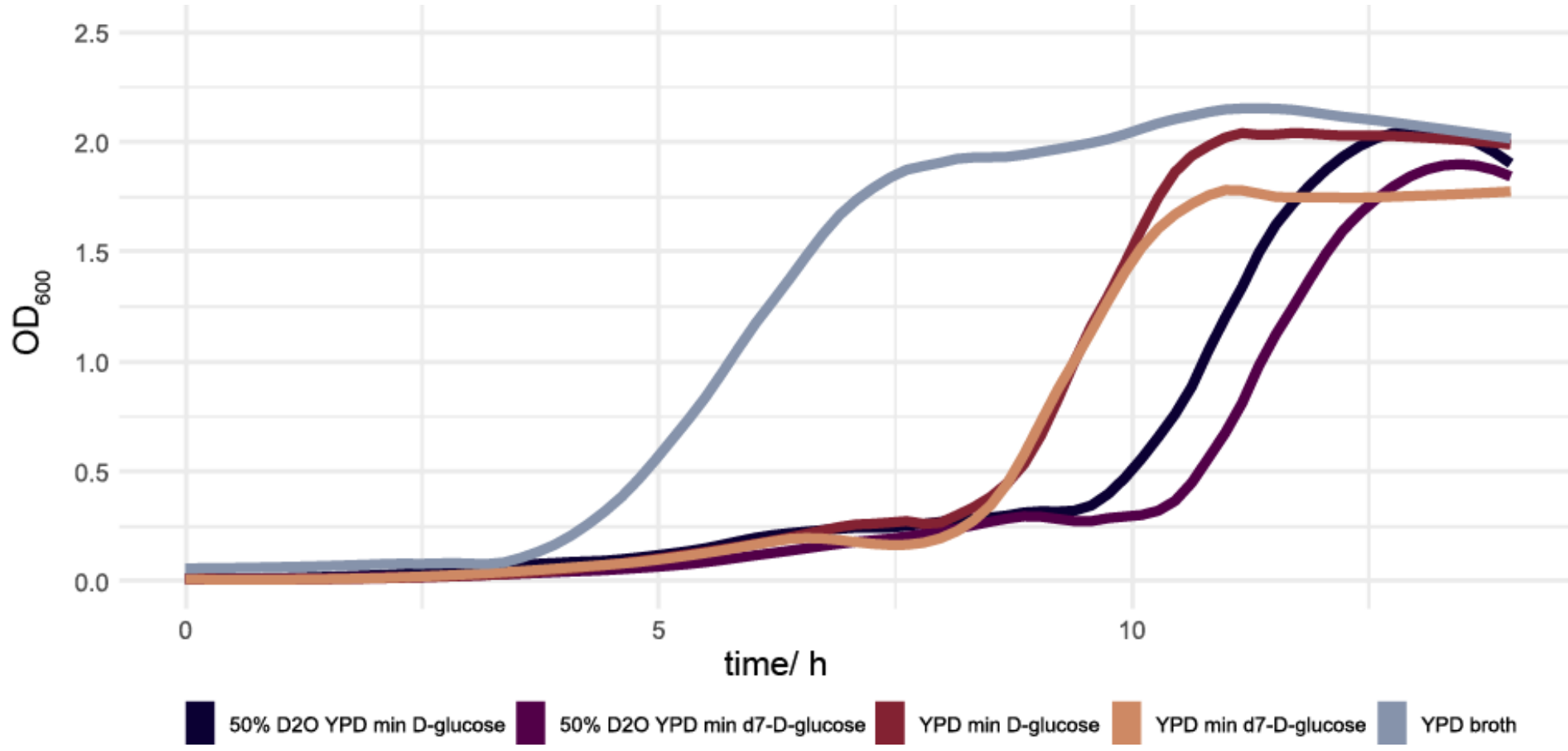

Figure S8. The growth curves with measured OD at 600 nm vs time of the *C. albicans* cells in different YPD based media at +30 °C.

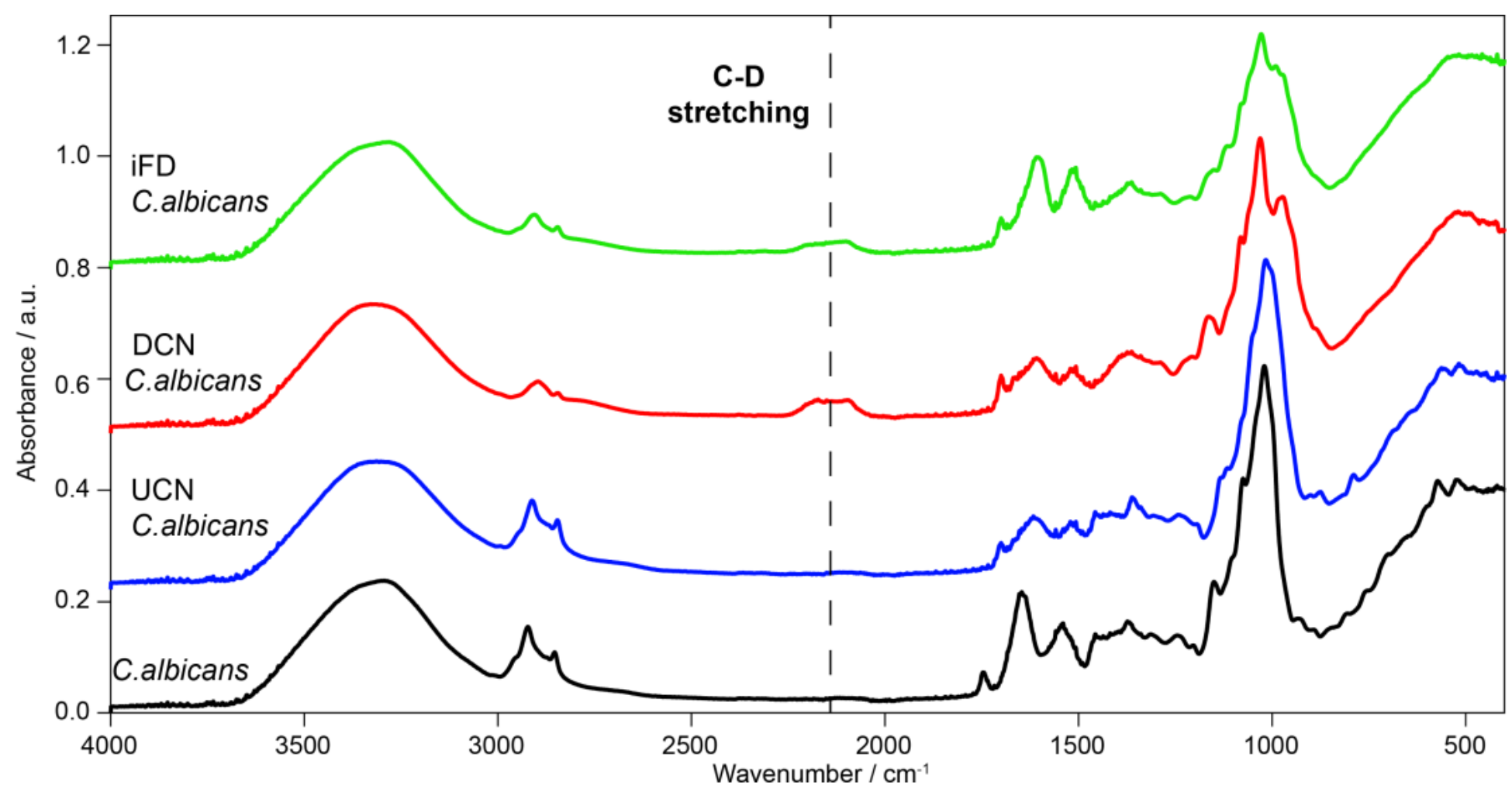

Figure S9. The overlay of ATR-FTIR spectra for protonated and deuterated *C. albicans* samples. The characteristic wavenumber of the C-D bond stretching is indicated with a dashed vertical line.

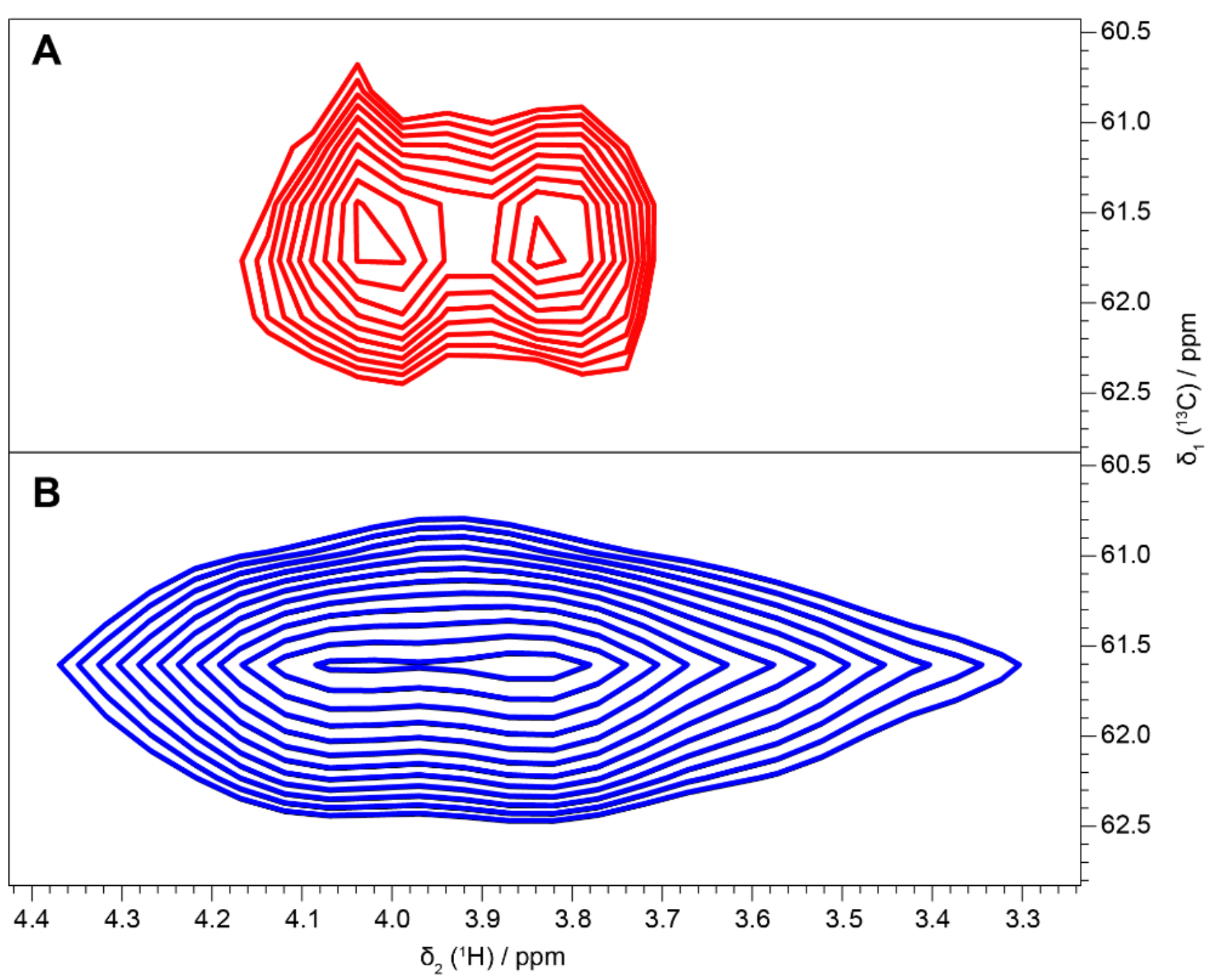

Figure S10. The 2D CP hCH spectral excerpts for $-CH_2-$ group in β-1,3-glucan at 150 kHz MAS. A - iFD, B - UCN.

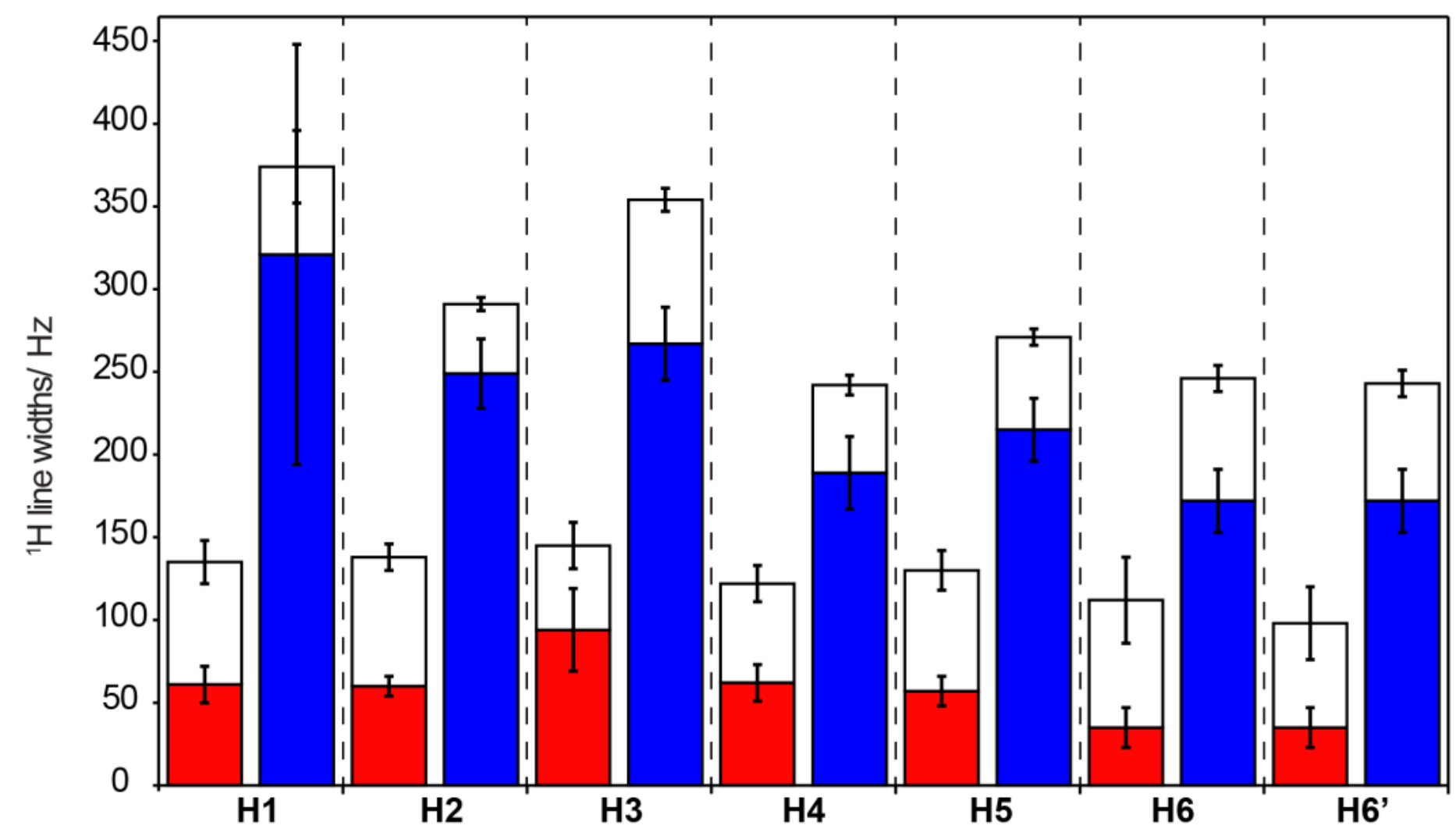

Figure S11. The homogeneous (red bars for 50%DCN and blue bars for UCN) and inhomogeneous (white bars) ^{1}H line widths for β-1,3-glucan in *C. albicans* at 150 kHz MAS. All relaxation time errors were expressed as 95% confidence intervals derived from the uncertainty of the exponential fit to the individual decay curves. Relative ^{1}H T'_2 relaxation time errors were used to calculate errors in ^{1}H linewidth.

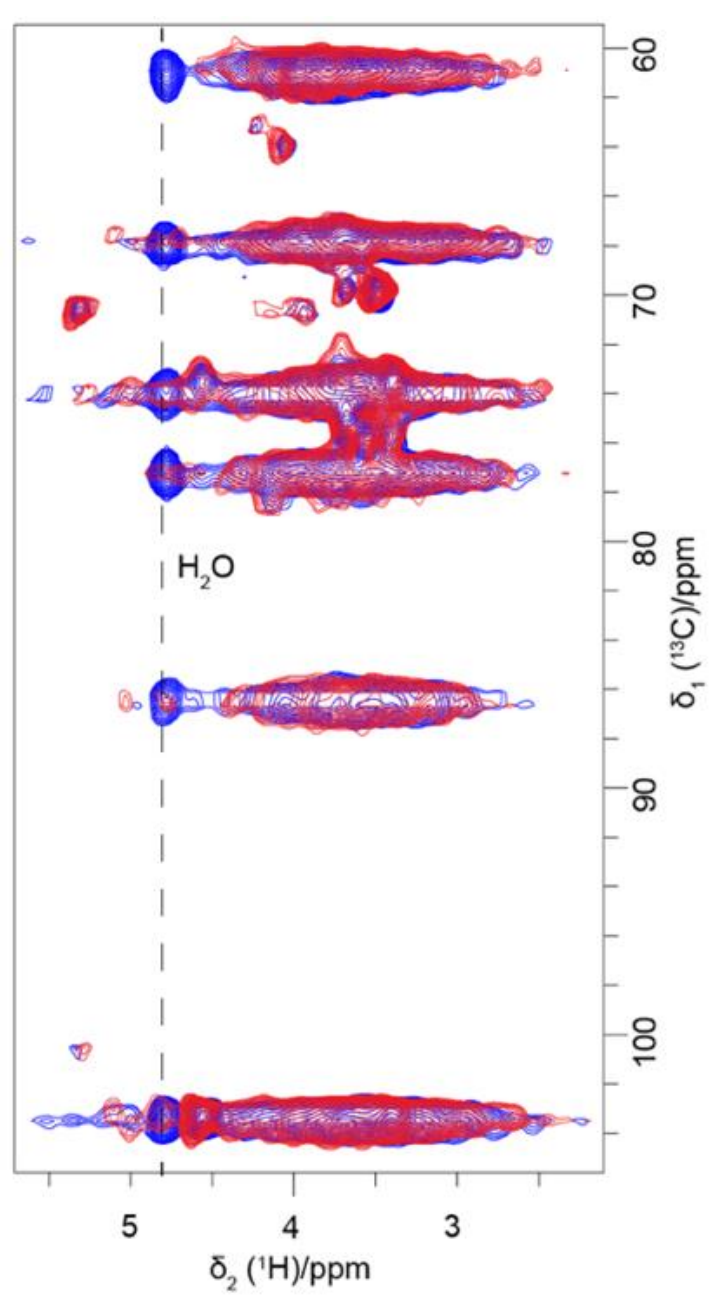

Figure S12. Overlay of 2D CP hCH spectra with a 3 ms long CP contact time for the UCN sample washed with H_2O (blue) and washed with D_2O (red) *C. albicans* samples. The sample washed with D_2O exhibited lower-intensity C-OH correlation peaks relative to H_2O. Both spectra were acquired at 40 kHz MAS in 1.3 mm rotors.

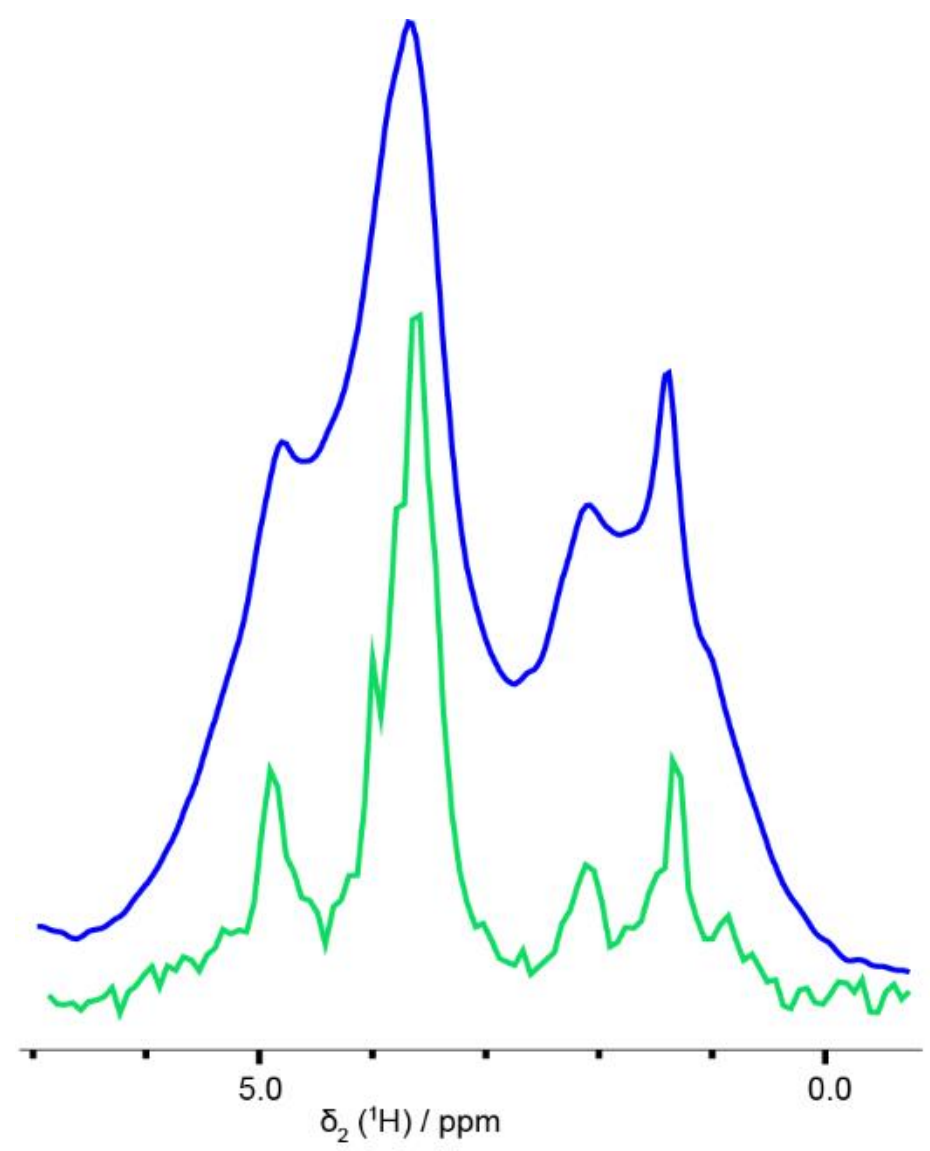

Figure S13.Overlay of 1D CP hCH spectra for UCN (blue) and iFD (green) *C. albicans* samples, acquired at 55.555 kHz MAS.

Table S1. The assigned ^{1}H and ^{13}C chemical shifts in ppm detected rigid glycans at 150 kHz MAS in CP-based experiments for the UCN *C. albicans* sample.

Glycans	H1/C1	H2/C2	H3/C3	H4/C4	H5/C5	H6H6'/C6	$-CH_3$
β-1,3-glucan	4.82<br>103.90	3.56<br>74.57	3.55<br>87.07	3.48<br>68.48	3.40<br>77.61	3.86<br>3.68<br>61.49	
Chitin		3.80<br>55.70					2.07<br>23.04

Table S2. Best-fit quadrupolar tensor parameters for ^{2}H-labeled glucose and iFD *C. albicans*.

Samples	C_Q [kHz]	η
^{2}H-Glucose	161.5±0.5	$0.01^{+0.03}_{-0.01}$
iFD *C. albicans*	89±2	$0.98^{+0.02}_{-0.06}$

Table S3. Experimental ssNMR parameters for 2D spectra.

Experiment	2D CP hCH (UCN)	2D INEPT hCH (UCN)	2D CP hCH (UCN)	2D 20 ms DARR (UCN)	2D CP hCH (iFD)	2D CP hCH (50%DCN)	2D CP hCH (50%DCN)
MAS frequency/kHz	150	150	60	12	60	60	150
Field/T	18.8	18.8	18.8	18.8	18.8	18.8	18.8
Transfer I	HC-CP	HC-INEPT	HC-CP	HC-CP	HC-CP	HC-CP	HC-CP
^{1}H field/kHz	117		143.3	60	143.3	143.3	191
^{13}C field/kHz	33		83.3	49	83.3	83.3	91
Shape	ramp ^{1}H		ramp ^{1}H	ramp ^{1}H	ramp ^{1}H	ramp ^{1}H	ramp ^{1}H
Time/ms	0.8	1.4	0.5	0.4	0.4	0.4	0.5
^{13}C carrier/ppm	60	60	60	74	60	60	60
Transfer II	CH-CP	CH-INEPT	CH-CP	DARR	CH-CP	CH-CP	CH-CP
^{1}H field/kHz	117		143.3	12	143.3	143.3	143.3
^{13}C field/kHz	33		83.3		83.3	83.3	83.3
Shape	ramp ^{1}H		ramp ^{1}H		ramp ^{1}H	ramp ^{1}H	ramp ^{1}H
Time/ms	0.8	1.4	0.6	20	0.4	0.4	0.6
^{1}H decoupling	slTPPM	slTPPM	slTPPM	SPINAL64	slTPPM	slTPPM	slTPPM
^{1}H decoupling field/ kHz	25	25	25	90	25	25	25
t1 increments	600	500	300	392	400	300	400
Window function	QSine 3	QSine 3	QSine 3	QSine 2	QSine 3	QSine 3	QSine 3
Sweep width (t1)/kHz	37	6.9	37	19.6	37	37	37
Acquisition time (t1)/ms	9.9	9.2	9.9	9.9	9.9	9.9	9.9
^{15}N/^{13}C decoupling	WALTZ16	WALTZ16	WALTZ16		WALTZ16	WALTZ16	WALTZ16
^{15}N/^{13}C decoupling field/ kHz	25	4	25	90	25	25	25
t2 increments	2048	4096	2048	2216	2048	2048	2048
Window function		QSine 3		QSine 2			
Sweep width (t2)/kHz	81.5	48.1	81.5	55.5	81.5	81.5	81.5
Acquisition time (t2)/ms	12.6	42	12.6	19.9	12.6	12.6	12.6
Interscan delay/s	1.7	1.2	1.7	3	1.7	1.7	1.7
Number of scans	16	16	16	96	32	16	32
Measurement time/h	5	2.8	2.2	21	6	2.2	6

Table S4. Experimental ssNMR parameters for 3D spectra acquired for the UCN *C. albicans* sample.

Experiment	3D CP hCCH TOCSY	3D CP HCcH TOCSY
MAS frequency/kHz	150	150
Field/T	18.8	18.8
Transfer I	HC-CP	HC-CP
^{1}H field/kHz	117	117
^{13}C field/kHz	33	33
Shape	ramp ^{1}H	ramp ^{1}H
Time/ms	0.5	0.4
^{13}C carrier/ppm	70	70
Transfer II	TOCSY	TOCSY
^{13}C field/kHz	35	35
Time/ms	14	14
Transfer III	CH-CP	CH-CP
^{1}H field/kHz	117	117
^{13}C field/kHz	34	34
Shape	ramp ^{1}H	ramp ^{1}H
Time/ms	0.6	0.4
t1 increments	130	80
Window function	QSine 3	QSine 2
Sweep width (t1)/kHz	20.1	8
Acquisition time (t1)/ms	3.2	4.9
t2 increments	130	100
Window function	QSine 2	QSine 2
Sweep width (t2)/kHz	20.1	18.1
Acquisition time (t2)/ms	3.2	2.8
t3 increments	2048	2048
Window function	QSine 3	QSine 3
Sweep width (t3)/kHz	81.5	81.5
Acquisition time (t3)/ms	12.5	12.5
Interscan delay/s	2	1.8
Number of scans	4	8
Measurement time/h	37.6	32

References

[1] I. Bertini, C. Luchinat, G. Parigi, E. Ravera, "SedNMR: On the Edge between Solution and Solid-State NMR" *Acc. Chem. Res.* **2013**, *46*, 2059–2069.
[2] A. Böckmann, C. Gardiennet, R. Verel, A. Hunkeler, A. Loquet, G. Pintacuda, L. Emsley, B. H. Meier, A. Lesage, "Characterization of different water pools in solid-state NMR protein samples" *J Biomol NMR* **2009**, *45*, 319–327.
[3] W. F. Vranken, W. Boucher, T. J. Stevens, R. H. Fogh, A. Pajon, M. Llinas, E. L. Ulrich, J. L. Markley, J. Ionides, E. D. Laue, "The CCPN data model for NMR spectroscopy: Development of a software pipeline" *Proteins* **2005**, *59*, 687–696.
[4] R. Aleksis, J. P. Carvalho, A. Jaworski, A. J. Pell, "Artefact-free broadband 2D NMR for separation of quadrupolar and paramagnetic shift interactions" *Solid State Nuclear Magnetic Resonance* **2019**, *101*, 51–62.
[5] M. Bak, J. T. Rasmussen, N. C. Nielsen, "SIMPSON: A General Simulation Program for Solid-State NMR Spectroscopy" *Journal of Magnetic Resonance* **2000**, *147*, 296–330.
[6] S. K. Zaremba, "Good lattice points, discrepancy, and numerical integration" *Annali di Matematica* **1966**, *73*, 293–317.

[7] H. Conroy, “Molecular Schrödinger Equation. VIII. A New Method for the Evaluation of Multidimensional Integrals” *The Journal of Chemical Physics* **1967**, *47*, 5307–5318.
[8] V. B. Cheng, H. H. Suzukawa, M. Wolfsberg, “Investigations of a nonrandom numerical method for multidimensional integration” *The Journal of Chemical Physics* **1973**, *59*, 3992–3999.
